# Translatome analysis of tuberous sclerosis complex 1 patient-derived neural progenitor cells reveals rapamycin-dependent and independent alterations

**DOI:** 10.1186/s13229-023-00572-3

**Published:** 2023-10-25

**Authors:** Inci S. Aksoylu, Pauline Martin, Francis Robert, Krzysztof J. Szkop, Nicholas E. Redmond, Srirupa Bhattacharyya, Jennifer Wang, Shan Chen, Roberta L. Beauchamp, Irene Nobeli, Jerry Pelletier, Ola Larsson, Vijaya Ramesh

**Affiliations:** 1 Department of Oncology-Pathology, Science for Life Laboratory, Karolinska Institute, 171 77 Stockholm, Sweden; 2 Center for Genomic Medicine, Department of Neurology, Massachusetts General Hospital, 185 Cambridge Street, Boston, MA 02114 USA; 3 Department of Biochemistry and Goodman Cancer Research Institute, McGill University, Montreal, PQ H3G1Y6 Canada; 4 Institute of Structural and Molecular Biology, Department of Biological Sciences,, Birkbeck, University of London, London, WC1E 7HX UK

**Keywords:** Tuberous sclerosis complex, TSC1, Neural progenitor cells, Translatome, Polysome profiling, Autism spectrum disorder, Early neurodevelopment, mTORC1, RMC-6272

## Abstract

**Background:**

Tuberous sclerosis complex (TSC) is an inherited neurocutaneous disorder caused by mutations in the *TSC1* or *TSC2* genes, with patients often exhibiting neurodevelopmental (ND) manifestations termed TSC-associated neuropsychiatric disorders (TAND) including autism spectrum disorder (ASD) and intellectual disability. Hamartin (TSC1) and tuberin (TSC2) proteins form a complex inhibiting mechanistic target of rapamycin complex 1 (mTORC1) signaling. Loss of TSC1 or TSC2 activates mTORC1 that, among several targets, controls protein synthesis by inhibiting translational repressor eIF4E-binding proteins. Using TSC1 patient-derived neural progenitor cells (NPCs), we recently reported early ND phenotypic changes, including increased cell proliferation and altered neurite outgrowth in *TSC1*-null NPCs, which were unaffected by the mTORC1 inhibitor rapamycin.

**Methods:**

Here, we used polysome profiling, which quantifies changes in mRNA abundance and translational efficiencies at a transcriptome-wide level, to compare CRISPR-edited *TSC1*-null with CRISPR-corrected *TSC1*-WT NPCs generated from one TSC donor (one clone/genotype). To assess the relevance of identified gene expression alterations, we performed polysome profiling in postmortem brains from ASD donors and age-matched controls. We further compared effects on translation of a subset of transcripts and rescue of early ND phenotypes in NPCs following inhibition of mTORC1 using the allosteric inhibitor rapamycin versus a third-generation bi-steric, mTORC1-selective inhibitor RMC-6272.

**Results:**

Polysome profiling of NPCs revealed numerous TSC1-associated alterations in mRNA translation that were largely recapitulated in human ASD brains. Moreover, although rapamycin treatment partially reversed the TSC1-associated alterations in mRNA translation, most genes related to neural activity/synaptic regulation or ASD were rapamycin-insensitive. In contrast, treatment with RMC-6272 inhibited rapamycin-insensitive translation and reversed TSC1-associated early ND phenotypes including proliferation and neurite outgrowth that were unaffected by rapamycin.

**Conclusions:**

Our work reveals ample mRNA translation alterations in TSC1 patient-derived NPCs that recapitulate mRNA translation in ASD brain samples. Further, suppression of TSC1-associated but rapamycin-insensitive translation and ND phenotypes by RMC-6272 unveils potential implications for more efficient targeting of mTORC1 as a superior treatment strategy for TAND.

**Supplementary Information:**

The online version contains supplementary material available at 10.1186/s13229-023-00572-3.

## Background

Tuberous sclerosis complex (TSC) is an inherited multisystem disorder, comprising both TSC1 and TSC2, and involves a range of symptoms including epilepsy, autism spectrum disorder (ASD), intellectual disability (ID) and slow growing hamartomas in many organs. TSC is caused by mutations in the *TSC1* or *TSC2* genes, encoding the tumor suppressor proteins hamartin (TSC1) and tuberin (TSC2) [1, 2]. The TSC proteins act as a central hub relaying signals from diverse cellular pathways to control mammalian/mechanistic target of rapamycin complex 1 (mTORC1) activity, which regulates cell growth and proliferation [3, 4]. Aberrant activation of mTORC1 in TSC has led to rapamycin (Rap) analogs (“rapalogs”) emerging as a lifelong therapy for TSC hamartomas, as their discontinuation leads to resumed growth of TSC-associated lesions [5–8]. Recent clinical trials revealed that rapalogs reduce epilepsy in 40% of TSC patients [9]. In contrast, rapalogs are ineffective in treating TSC-associated neuropsychiatric defects (TAND) and autism [10, 11]. Therefore, new treatments for TSC that are superior to rapalogs with respect to anti-proliferative effects in tumors, and efficacy toward the non-tumor CNS manifestations of the disorder are needed.

Among mTOR inhibitors, first-generation allosteric rapalogs effectively suppress phosphorylation of mTORC1 target S6K1, but not 4E-BP1 in many cell types [12, 13]. Furthermore, allosteric rapalogs activate AKT, a downstream target of mTORC2, by negative feedback loops [14], which prompted development of second-generation, orthosteric mTOR kinase inhibitors (active site mTOR inhibitors) including Torin 1, AZD8055 and TAK-228/MLN0128, which potently inhibit both mTORC1 and mTORC2. As mTORC2 promotes lipogenesis, glucose uptake and cell survival through downstream targets AKT and SGK, active site mTOR inhibitors appears to be more toxic than rapalogs [12, 15]. The limited clinical benefits of first- and second-generation mTOR inhibitors led to the recent development of a third-generation mTORC1-directed inhibitor RapaLink-1. This bi-steric mTOR inhibitor links the high affinity of rapamycin for mTORC1 with the effective active site mTOR inhibition of TAK-228 [16]. RapaLink-1 was shown to be highly potent in reducing phosphorylation of both S6K1 and 4E-BP1 while retaining approximately four-fold selectivity for mTORC1 as compared to mTORC2. More recent bi-steric compounds such as RMC-6272 show higher selectivity for mTORC1 over mTORC2 (more than 30-fold selectivity), along with potent suppression of 4E-BP1 phosphorylation [17, 18].

Several mouse models of TSC have provided valuable clues regarding neurological symptoms, but incompletely recapitulate the human phenotypes [19]. Recent studies examining the role of TSC1 or TSC2 have employed genetically engineered human embryonic stem cell lines with heterozygous or homozygous loss of *TSC2*; TSC patient-derived induced pluripotent stem cells (iPSCs); or isogenic gene-edited iPSCs from patients with *TSC1* or *TSC2* mutations that have been differentiated into, e.g., neural progenitor cells (NPCs), forebrain neurons, cerebellar Purkinje neurons, astrocytes or oligodendrocytes [20–28]. Many phenotypic alterations, including somatic hypertrophy, increased dendritic arborization, augmented proliferation rate, altered electrophysiology and hyperactivation of mTORC1, are more pronounced in *TSC1*-null or *TSC2*-null cells when compared with heterozygous or wild-type (WT) counterparts (reviewed in [29, 30]). Furthermore, transcriptome analyses have revealed ample alterations when comparing isogenic gene-edited *TSC1-* or *TSC2*-null NPCs or neurons to heterozygous or WT cells [21, 22, 24, 26].

Among its many activities [14], mTORC1 plays a major role in regulating gene expression by modulating how efficiently mRNAs are translated into proteins [31]. Consistent with a key role of mRNA translation in determining proteome composition, translatomes (commonly defined as the pool of mRNA associated with ribosomes) [32] resemble proteomes more closely than corresponding transcriptomes [33–35]. mTORC1 regulates cap-dependent translation by modulating the assembly of eukaryotic initiation factor (eIF) 4F, a complex consisting of a cap binding protein (eIF4E), a DEAD-box RNA helicase (eIF4A) and a large scaffolding protein (eIF4G). mTORC1 activation leads to direct phosphorylation of two key substrates involved in regulating translation initiation: eIF4E-binding proteins (4E-BPs) and ribosomal protein p70S6 kinases (S6Ks). 4E-BPs are a family of translation inhibitors consisting of three members, the best studied being 4E-BP1, which when de-phosphorylated competes with eIF4G for binding to eIF4E and prevents eIF4F complex formation. Once phosphorylated, 4E-BP1 dissociates from eIF4E, facilitating eIF4F complex formation [36]. Further, activation of S6K by mTORC1 also affects translation initiation by: (1) increasing eIF4A availability through phosphorylation and degradation of a negative repressor, PDCD4, and (2) phosphorylating eIF4B which stimulates eIF4A helicase activity and promotes initiation complex formation [37, 38]. Interestingly, a recent study reported that mTORC1-dependent translation is high in human pluripotent stem cells and is suppressed during neural differentiation [39]. Moreover, numerous changes in mRNA translation without corresponding changes in mRNA levels have been observed across human neuronal development, highlighting the importance of translational control for developing neurons [39].

Our comparisons of transcriptomes between isogenic NPCs revealed a quantitative genotype-dependent response whereby genes upregulated/downregulated in *TSC1*-heterozygous NPCs were further increased/decreased in *TSC1*-null cells when compared to genetically matched CRISPR-corrected WT cells. Interestingly, this included genes linked to ASD, epilepsy and ID [24]. However, despite alterations in mRNA translation being a major mechanism modulating gene expression downstream of mTOR in cancer cells [40], translatome studies are lacking in TSC stem cell models. Recent studies have documented that early neurodevelopmental events, such as NPC proliferation, neurite outgrowth and migration, that precede synaptogenesis also play a role in disease pathogenesis of ASD and other neuropsychiatric disorders [41–47]. The enhanced proliferation and neurite outgrowth consistently observed in *TSC1*-null NPCs when compared with isogenic WT controls suggest that this may underlie early neurodevelopmental defects in TSC [24].

Using our isogenic NPC model generated from TSC1 patient-derived iPSCs, we identified TSC1-sensitive mRNA levels and translation. Strikingly, TSC1-sensitive mRNA translation observed in NPCs was recapitulated in human ASD brain samples from the Brodmann area 19 when contrasted to controls. Furthermore, although polysome profiling revealed a partial reversal of TSC1-sensitive translation upon rapamycin treatment, most genes related to neural activity/synaptic regulation or ASD showed rapamycin-insensitive translation. However, translation of a subset of rapamycin-insensitive genes could be reversed by the mTORC1-selective inhibitor RMC-6272, which more efficiently suppresses 4E-BP1-phosphorylation in NPCs when compared to rapamycin [18]. This was accompanied by reversal of rapamycin-insensitive phenotypes in *TSC1*-null NPCs, suggesting that more efficient targeting of mTORC1 may be an attractive treatment strategy in ASD.

## Methods

### Cell lines and reagents

NPCs derived from one female TSC1 patient donor, including *TSC1*-het (+/-) parental cell line, as well as CRISPR-deleted *TSC1*-null (−/−) and CRISPR-corrected *TSC1*-WT (+/+) lines (one clone/genotype), along with culture conditions, have been previously described [17]. Rapamycin was from EMD Millipore (Burlington, MA), and RMC-6272 (previously known as RM-006) was generously provided by Revolution Medicines, Inc. (Redwood City, CA). All antibodies are listed in Additional file 2: Table S1.

### Polysome fractionation and RNA sequencing

Lysate preparation for polysome profiling from three biological replicates was carried out as previously described [48]. Briefly, TSC1 NPC lines were seeded at 40,000 cells/cm^2^, with a total of 5 × 10^7^ cells seeded per drug treatment condition. The next day after seeding, cells were treated for 2 h with 50 nM rapamycin, 10 nM RMC-6272 or DMSO as a vehicle control. Treated cells were then rinsed with 1X PBS containing 100 µg/ml cycloheximide (CHX) (Sigma, St. Louis, MO), harvested by scraping on ice in PBS/CHX and pelleted by centrifugation at 300*g* for 10 min at + 4C. Cell pellets were lysed in 5 mM Tris–HCl (pH 7.5), 1.5 mM NaCl, 2.5 mM MgCl_2_, 0.5% sodium deoxycholate, 0.5% Triton-X-100, 2 mM DTT and 100 μg/ml CHX. Lysates were cleared by spinning for 2 min at 13,000 rpm and quickly frozen on dry ice. When ready for processing, lysates were thawed and loaded onto a 10–50% sucrose gradient, centrifuged for 2 h and 15 min at 35,000 rpm in a SW41 rotor using a Sorvall Discovery 90SE centrifuge. The gradients were fractionated on a Teledyne ISCO Foxy R1 apparatus while monitoring the OD_254nm_. Fractions corresponding to mRNA associated with more than two ribosomes were pooled and the RNA extracted using TRIzol (Thermo Fisher, Waltham, MA) according to the manufacturer’s protocol. Prior to loading samples on the sucrose gradient, RNA was extracted from 10% of the lysate using TRIzol, and the resulting RNA was denoted as total RNA. RNA sequencing libraries were prepared from the resulting samples using Illumina v2.5 Kits and sequenced (three biological replicates) on an Illumina NextSeq 500 at the Canada's Michael Smith Genome Sciences Centre (BC Cancer Research Institute, Vancouver, Canada).

### Polysome fractionation of postmortem brain samples

Polysome fractionation of postmortem samples from the Brodmann area 19 (BA19) brain region from ASD-affected donors (*n* = 6) and matched controls (*n* = 4) provided by NIH NeuroBiobank was performed using an optimized sucrose gradient, as previously described [49, 50]. For included donors, there is only incomplete information of comorbidities, medications and severity. Moreover, RNA sequencing data did not support *TSC1*-mutations in any of the ASD donors. RNA sequencing libraries were generated using the Smart-seq2 protocol as described previously [50]. Single-end 51 base sequencing was performed using the HiSeq2500 platform and the HiSeq Rapid SBS kit v2 chemistry at the National Genomics Infrastructure, Science for Life Laboratory, Stockholm, Sweden. Bcl to fastq conversion was performed using bclfastq_v2.19.1.403 from the CASAVA suite.

### RNA-Seq data preprocessing and quality control

Quality of sequencing reads (paired-end for NPC dataset; single-end for postmortem brain dataset) was confirmed using FastQC (v0.11.4; http://www.bioinformatics.babraham.ac.uk/projects/fastqc/). BBmap (v. 36.59: https://www.osti.gov/servlets/purl/1241166; parameters: k = 13, ktrim = n, useshortkmers = t, mink = 5, qtrim = t, trimq = 10, minlength = 25) was used to trim reads for Illumina Truseq and Nextera adapter sequences and low-quality base calls. For the NPC dataset, BBmap (v. 36.59) was used to remove sequencing reads mapping to rRNA sequences obtained from the SILVA ribosomal RNA gene database [51]. The resulting reads were aligned to the human reference genome (build hg38) using HISAT2 (v.2.1.0 and v.2.0.4 for NPC and postmortem brain datasets, respectively, using, in addition to default parameters, “–no-mixed” and “–no-discordant” parameters for the NPC dataset) [52]. The aligned reads were summarized using the “featureCounts” function of the RSubread (v.2.6.4) R/Bioconductor package [52] and the reads were assigned using the hg38 GTF annotation from the UCSC database [53] (parameters: isPairedEnd = isPairedEnd = autosort = T, allowMultiOverlap = F, strandSpecific = 2 for NPC dataset and ignoreDup = FALSE, useMetaFeatures = TRUE, countMultiMappingReads = FALSE for postmortem brain dataset). A summary of the read counts at each preprocessing step was plotted using ggplot2 (v.3.3.6) (Additional file 1: Fig. S1A and Fig. S2A). Expression of TSC1 was further assessed in the NPC dataset using TMM-log2 normalized counts obtained by running calcNormFactors function from edgeR (v.3.34.1) [50] and voom function from limma (v.3.48.3) [52] (Additional file 1: Fig. S1B). Principal component analysis (PCA) on normalized counts [50] was performed using the PCAtools R package (v. 2.4.0; https://github.com/kevinblighe/PCAtools; parameters removeVar = 0.75 and scale = T) and visualized using eigencorplot, screeplot and biplot functions from the PCAtools R package (v.2.4.0; https://ggplot2.tidyverse.org; Additional file 1: Fig. S1C-E and Fig. S2B-D).

### Analysis of gene expression alterations using anota2seq

Genes with 0 mapped RNA sequencing read in one or more samples were discarded resulting in analysis of 12,950 and 11,998 genes in postmortem brain samples and NPCs, respectively. The data were TMM-log2 normalized and analyzed using anota2seq [54] [v. 1.14.0; parameters: minSlopeTranslation = − 1, minSlopeBuffering = − 2, maxSlopeTranslation = 2, maxSlopeBuffering = 1, deltaPT = deltaTP = deltaP = deltaT = log2(1.2)] [54, 55]. During analysis of the NPC dataset, replicate was included in the model to correct for batch effects and three contrasts were assessed, namely (1) *TSC1*^*−/−*^ versus *TSC1*^+*/*+^, (2) *TSC1*^*−/−*^ + rapamycin versus *TSC1*^*−/−*^ and (3) *TSC1*^+*/*+^  + rapamycin versus *TSC1*^+*/*+^, with the following thresholds to identify differentially expressed genes: minEff = log2(1.5) and maxRvmPadj = 0.15 [i.e., fold change > log2(1.5) and FDR < 0.15]. In the postmortem dataset, 6 ASD-affected brains were compared to 4 neurotypical, and relaxed thresholds were applied: minEff = log2(1.25) and pVal = 0.05.

### Gene ontology analysis

Genes identified as regulated via the “translation” mode in anota2seq (i.e., transcripts with an increase or decrease in polysome-associated mRNA levels without corresponding changes in total cytosolic mRNA levels) were used as input for gene set enrichment analysis using Cytoscape (v.3.8.2.) plug-in ClueGO (v.2.5.8) [56] with FDR cutoff = 0.001 or 0.01 for NPCs and postmortem brain samples, respectively, together with p value cutoff = True, Correction Method Used = Benjamini–Hochberg, Statistical Test Used = Enrichment (Right-sided hypergeometric test), Kappa = 0.4, Min. Percentage = 10, Min GO Level = 7, Max GO Level = 15, Number of Genes = 3, GO Fusion = false, GO Group = true, Over View Term = SmallestPValue, Group By Kapp Statistics = true, Initial Group Size = 1, Sharing Group Percentage = 50.0, Ontology Used = GO_BiologicalProcess-EBI-UniProt-GOA-ACAP ARAP_13.05.2021_00h00, KEGG_13.05.2021, REACTOME_Reactions_13.05.2021, Evidence codes used = All, Identifiers used = SymbolID. The resulting networks were set to show the most significant term identified for each group.

### Analysis of gene signatures using empirical cumulative distribution functions

To cross-compare RNA-seq datasets, empirical cumulative distribution functions (ECDFs) of log2 fold changes for polysome-associated and cytosolic mRNA were plotted for genes that were found to be translationally regulated in either dataset. The difference between each tested gene set and the background was quantified at the 50th quantile and the Wilcoxon rank-sum test was used to determine whether there was a significant shift between the background and each signature. The same approach was used to assess signatures of transcripts whose translation was previously identified as increased upon eIF4E overexpression [57] or genes associated with synaptic function [58].

### NanoString nCounter Gene Expression Analysis

#### Target gene selection and generation of custom NanoString panel

For NanoString nCounter analysis [59], a custom panel of 200 target genes identified by anota2seq analysis as regulated by “translation” or “mRNA abundance” were selected: (1) genes with log2FC > 2 and FDR < 0.15 in any of the contrasts applied when analyzing the NPC dataset, (2) targets annotated to ASD/NDD pathology with log2FC > 1 and FDR < 0.15 in the NPC dataset and (3) negative controls were identified based on standard deviation (< 0.3) between samples, mean TMM-log2 signal in the top 50th quartile, deltaT < 0.1 (from anota2seq analysis) and deltaP < 0.1 (from anota2seq analysis).

#### NanoString analysis

Samples from each condition were randomized on cartridges and processed by the KIGene Core Facility (Karolinska Institute, Sweden) using 100 ng input for cytosolic mRNA and 300 ng input for polysome-associated mRNA. The “newRccSet” function from the NanoStringQCPro (v.1.24.0) R/Bioconductor package was used to preprocess raw data. Genes with expression less than 6.27 (log2 scale; *TSC1*^*−/−*^ vs *TSC1*^+*/*+^ comparison) and 5.63 (log2 scale; *TSC1*^*−/−*^ + Rap vs *TSC1*^*−/−*^ and *TSC1*^*−/−*^ + RMC-6272 vs *TSC1*^*−/−*^ comparisons) in 3 or more samples were excluded [thresholds were determined by calculating the mean log2 expression level of negative control genes + 2 standard deviation (SD)]. This resulted in analysis of 175 genes for the *TSC1*^*−/−*^ versus *TSC1*^+*/*+^ comparison while 164 transcripts were included in *TSC1*^*−/−*^ + Rap versus *TSC1*^*−/−*^ and *TSC1*^*−/−*^ + RMC-6272 versus *TSC1*^*−/−*^ comparisons. For the *TSC1*^*−/−*^ versus *TSC1*^+*/*+^ comparison, the geNorm function of the CtrlGene (v.1.0.1) R/Bioconductor package identified *BUD31, BASP1*, and *GAB2* as housekeeping genes for normalization and data were normalized using the contentNorm function (from the NanoStringQCPro package) with the following parameters: method = “housekeeping,” summaryFunction = “mean” and hk = *(BUD31, BASP1*, *GAB2)*. An additional step of variance stabilizing normalization (vsn) was then performed using the justvsn function from the vsn(v.3.60.0) [60] R/Bioconductor package. For the *TSC1*^*−/−*^ + Rap versus *TSC1*^*−/−*^ and *TSC1*^*−/−*^ + RMC-6272 versus *TSC1*^*−/−*^ comparisons, no housekeeping genes could be identified (possibly as the RMC-6272 treatment was not included during selection of housekeeping genes) Therefore, global normalization was performed using the contentNorm function with the following parameters: method = “housekeeping,” summaryFunction = “mean.” Similar to above, vsn normalization was then performed. Log2 fold changes were then calculated and plotted.

### Validation of differential translation using RT-qPCR

To validate using RT-qPCR, polysomes from *TSC1*^−/−^ or *TSC1*^+*/*+^ NPCs (three biological replicates) were fractionated and pooled as described for NPCs above. RNA was isolated using TRIzol (ThermoFisher, Waltham, MA) and cDNA was prepared using M-MuLV Reverse Transcriptase (New England Biolabs, Ipswich, MA) and oligo(dT)20 primers using manufacturer’s recommendations. RT-qPCRs were performed with SsoFast Evagreen Supermix (Bio-Rad, Hercules, CA) using the CFX96 PCR system (Bio-Rad Hercules, CA). Primers for RT-qPCR are detailed in Additional file 3: Table S2. The level of each mRNA was normalized to the geometric mean of β-actin (*ACTB*), Phosphoglycerate Kinase 1 (*PGK1*) and Hypoxanthine Phosphoribosyl transferase 1 (*HPRT1*) using the comparative CT method and compared across conditions as indicated in figure legends.

### Cell size and proliferation analysis

For cell size and proliferation assays using trypan blue exclusion methods [61], three biological replicates per treatment (each including three technical replicates) were performed. Briefly, cells were rinsed in PBS and detached using Accutase enzyme detachment medium (ThermoFisher, Waltham, MA). Next, for each sample, 10 µl of suspended cells was combined with 10 µl of trypan blue, and 10 µl of the resulting mixture was added to two replicate chambers of a disposable Countess chamber slide and inserted into the Countess II automated cell counter (ThermoFisher, Waltham, MA), according to the manufacturer’s instructions. Quantitation of viable and dead cell counts as well as size of viable cells in microns, after exclusion of objects consistent with cellular debris, was performed. For each technical replicate, two repeat measurements, including counts of viable versus dead cells as well as size of viable cells, from the two chambers were averaged and recorded. Proliferation was also assessed using flow cytometry based on expression of the proliferation marker Ki-67 as well as real-time, cellular image-based analysis. For Ki-67-based methods, *TSC1*^+*/*+^ and *TSC1*^−/−^ NPCs were seeded onto Geltrex-coated 150 mm plates at 0.75 × 10^6^ cells/plate (two biological replicates per cell line, per treatment condition). Following overnight attachment, NPCs were treated with 50 nM rapamycin, 10 nM RMC-6272 or DMSO as vehicle control for 72 h. To assess the Ki-67 proliferation status, monoclonal AlexaFluor488-conjugated Ki-67 antibody (Cell Signaling Technologies, Danvers, MA) was employed and immunostaining was performed according to the manufacturer’s instructions. Briefly, NPCs were harvested using Accutase, pelleted, washed with PBS and fixed for 15 min at room temperature with 4% paraformaldehyde. Following fixation, cells were washed twice with PBS and permeabilized with ice cold 100% methanol to a final concentration of 90% with gentle vortexing and incubated for 30 min on ice. Thereafter, cells were stained using Ki-67 at 1:50 for 1 h, and data were acquired using a BD LSR II Flow Cytometer (BD Biosciences). For all treatment conditions, 1 × 10^4^ cells were acquired and recorded. Data analyses were carried out using FlowJo 10.8.1 (FlowJo LLC, Ashland, OR, USA). Percentage of Ki-67 positive cells (acquired through FITC channel) were determined by gating with respect to unstained control for both the *TSC1*^+*/*+^ and *TSC1*^−/−^ NPCs, respectively. For image-based methods, *TSC1*^+*/*+^ and *TSC1*^−/−^ NPCs were seeded at 0.3 × 10^5^ cells/well of a 24-well Geltrex-coated plate (three biological replicates per cell line, per treatment). The next day after seeding, medium was exchanged for fresh medium containing the fluorescent nuclear marker NucSpot650 (Biotium, Fremont, CA) at 1:500 dilution, according to the manufacturer’s instructions, along with 50 nM rapamycin, 10 nM RMC-6272 or DMSO as a vehicle control. Medium containing DMSO without NucSpot650 was used as an unstained control. Briefly, live, time-lapse images were acquired using an Incucyte SX5 (Sartorious, Göttingen, Germany) with the 10 × objective. Immediately after seeding, an initial image was acquired to measure the baseline confluence value. NucSpot650 and compounds were added 28 h following seeding, with images acquired every 2 h. The Phase channel was used to image cells, and the NIR channel was used to image nuclei stained with NucSpot650 with a 400 ms exposure. Image acquisition and analysis were performed with the Incucyte 2021C version of the software. We used the Basic Analyzer module to segment cells for the confluence metric and NucSpot650 for the nuclear count metric. Briefly, the parameters for the cell segmentation included a 1.1 Segmentation Adjustment and a minimum area filter set at 300 µm^2^, and NucSpot650 stained nuclei were segmented using a Top-Hat Segmentation with a radius of 20 µm and NIRCU Threshold value of 2. To filter out dead nuclei, a threshold for the mean max intensity was set at 50 NIRCU. For each well in a 24-well plate, 36 non-overlapping locations were imaged.

### Neurite outgrowth assays

For neurite outgrowth, NPCs (6250/cm^2^) were seeded on Poly-D-lysine coated wells (0.1 mg/ml; Sigma, St. Louis, MO) and Fibronectin (5 µg/ml, Corning, Corning, NY) in growth factor-depleted neural expansion medium (30% NEM) containing 1:1 of neurobasal media and advanced DMEM/F12 (ThermoFisher, Waltham, MA), 1 × penicillin/streptomycin and 0.3 × neural induction supplement (ThermoFisher, Waltham, MA). Cells were grown in the presence of DMSO, 50 nM rapamycin or 10 nM of RMC-6272 for 48 h and fixed with 4% paraformaldehyde (PFA; Microscopy Sciences, Hatfield, PA) for 20 min prior to immunostaining. Cover slips from three biological replicates were analyzed. For each cell line, images from eight independent non-overlapping fields/treatment condition were analyzed using HCA-Vision software V.2.2.0 (CSIRO, Canberra, Australia), which was developed to trace and quantify neurite structure in several parameters including: (1) number of cells; (2) average number of neurites/cell, defined as number of root points where neurites emerge from the cell body; (3) average neurite outgrowth/cell, defined as total length of all neurite structures including primary and branched outgrowths emerging from a body; and (4) average number of extremities/cell, defined as the number of termination points for all segment structures/cell [62]. Images were acquired on a Nikon Eclipse TE2000-U microscope using a Nikon DS-QiMc camera and NIS-Element BR 3.2 imaging software.

### Immunocytochemistry

Cells were fixed with 4% PFA for 20 min at room temperature and washed three times with PBS. Non-specific labeling was blocked, and cell membranes permeabilized in a single step, using 4% normal goat serum (NGS) in PBS containing 0.1% Triton-X-100 and 0.05% Tween-20 for 45 min at room temperature. Primary antibodies were diluted in 2% NGS/0.1%Triton-X-100/PBS and incubated for 2 h in the dark at room temperature (see Additional file 2: Table S1 for primary antibodies). Coverslips were mounted in ProLong Gold antifade reagent with DAPI (Invitrogen, Carlsbad, CA) and immunofluorescence was visualized on a Nikon Eclipse TE2000-U microscope. Images were acquired using a Nikon DS-QiMc camera and NIS-Element BR 3.2 imaging software.

### Immunoblot analyses

Immunoblotting was performed as previously described [24]. Briefly, cells were lysed in RIPA buffer, and protein lysates were resolved on Novex 4–12% or 10–20% Tris–Glycine gels (Invitrogen, Carlsbad, CA), transferred to nitrocellulose (Bio-Rad, Hercules, CA) and then incubated with primary antibodies (see Additional file 2: Table S1 for primary antibodies). All immunoblotting data shown are a representative of at least three biological replicates.

### Statistical analysis

For RNA sequencing, statistical analyses from three biological replicates were performed using RStudio (R v.4.1.1). Changes in translational efficiency were assessed using batch-adjusted analysis of partial variance (APV) in combination with a random variance model implemented in the anota2seq bioconductor package. The p values obtained from the analysis were adjusted using the Benjamini–Hochberg (BH) method. ECDFs were used to cross-compare RNA-seq datasets and assess selective regulation of signatures, and significance was assessed using the Wilcoxon rank-sum test relative to the background. Right-sided hypergeometric tests were used to identify GO terms enriched for genes identified by anota2seq with an FDR cutoff 0.001 or 0.01 for NPCs or postmortem brain samples, respectively (BH method). All tests were two-tailed unless otherwise indicated. For cell size and proliferation, p values were determined from three biological replicates by one-tailed Student’s t test. For neurite outgrowth assays, quantitation from three biological replicates was performed from using HCA-Vision software, and p values were calculated by one-tailed Student’s t test. Plots for neurite data were generated using GraphPad Prism9. Details related to each figure regarding iPSC-derived NPCs and number of independent experiments and replicates are shown in Additional file 8: Table S7.

## Results

### TSC1 loss leads to widespread alterations of mRNA translation in patient-derived isogenic neural progenitor cells (NPCs)

**Fig. 1:**
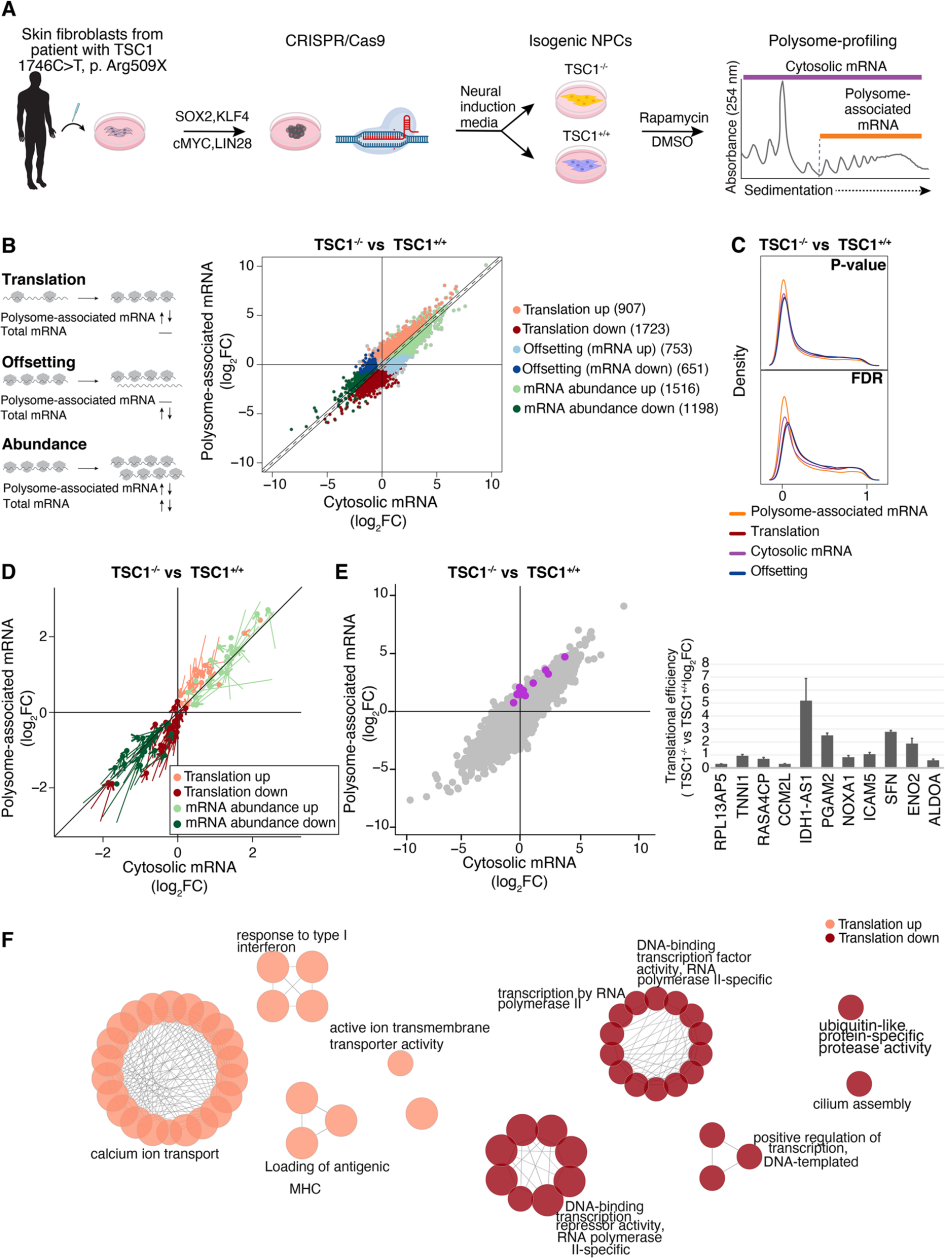
TSC1-associated alterations in mRNA abundance and translation. **A** Overview of the polysome-profiling approach in NPCs to identify TSC1-associated changes in gene expression. **B** Overview of modes for gene expression alterations identified by anota2seq where changes in mRNA levels and their ribosome association are indicated by curled lines (mRNA) and gray circles (ribosomes), respectively (left). Scatter plot from anota2seq analysis comparing polysome-associated to cytosolic mRNA log_2_ fold changes for the *TSC1*^*−*/−^ NPC versus *TSC1*^+/+^ NPC comparison (right). Genes identified as differentially regulated by anota2seq (see “Methods” section for applied thresholds) in each gene expression mode are visualized in the scatter, and the total number of genes is indicated in brackets. **C** Kernel densities of p values or FDRs (from anota2seq analysis) for the comparison of *TSC1*^−/−^ versus *TSC1*^+/+^ NPCs. Densities are shown for analysis of polysome-associated RNA, cytosolic RNA, translation and offsetting. **D** Scatter plot of log2 fold changes comparing *TSC1*^−/−^ versus *TSC1*^+/+^ NPCs estimated using NanoString nCounter assays. Each gene is represented by an arrow where the start of the arrow shows the fold changes estimated by RNA sequencing (i.e., **B**) and the end of the arrow indicates the fold change obtained by NanoString nCounter assays. **E** Scatter plot from anota2seq analysis comparing polysome-associated to cytosolic mRNA log_2_ fold changes for the *TSC1*^*−*/−^ NPC versus *TSC1*^+/+^ NPC comparison (left panel) showing genes randomly selected for validation by RT-qPCR (purple) relative to background (gray). The right panel indicates changes in translational efficiencies (i.e., polysome-associated mRNA normalized to cytosolic mRNA) according to RT-qPCR for the same genes. **F** Gene-set enrichment analysis for proteins encoded by mRNAs whose translation increase or decrease in *TSC1*^−/−^ versus *TSC1*^+/+^ NPCs. Each node corresponds to a process or pathway and edges connect nodes that were identified from overlapping genes (see “Methods” section for details)

To determine the impact of *TSC1* loss-of-function mutation on mRNA levels and translation in NPCs, we used skin fibroblasts from one female patient donor with a heterozygous mutation in *TSC1* exon 15 (1746C > T, Arg509X) to derive isogenic *TSC1*-null (−/−) NPCs and corrected *TSC1-*WT (+/+) NPCs (one CRISPR-edited clone/genotype) as described previously [24] (Fig. 1A and Additional file 1: Fig. S3A). This isogenic cell pair was then used to assess TSC1-associated changes in gene expression at multiple levels using polysome profiling [49]. During polysome profiling, total cytosolic mRNA is fractionated depending on ribosome association and a pool of polysome-associated mRNA is isolated in parallel with total cytosolic mRNA (Fig. 1A). The resulting RNA pools from *TSC1*^−/−^ and *TSC1*^+/+^ NPCs were quantified using RNA sequencing followed by analysis using anota2seq [54, 55] to identify three modes of TSC1-associated gene expression alterations: (1) changes in polysome-associated mRNA not paralleled by corresponding alterations in total mRNA levels (denoted “translation” and, under conditions when translation elongation is unaffected, interpreted as changes in translational efficiency leading to modulation of protein levels); (2) congruent changes of polysome-associated and total mRNA (denoted “abundance” representing alterations in mRNA levels impacting protein levels downstream of, e.g., modulation of transcription and/or mRNA-stability); and (3) alterations in total mRNA not paralleled by corresponding changes in polysome-associated mRNA (denoted “offsetting” and interpreted as instances where mRNA translation opposes alterations in protein levels imposed by modulation of mRNA levels [discussed in detail elsewhere] [63, 64]) (Fig. 1B, left). As visualized by a scatterplot comparing TSC1-associated changes in total cytosolic and polysome-associated mRNA (Fig. 1B, right), and densities of p values or FDRs (Fig. 1C) from anota2seq analysis, this revealed numerous TSC1-associated alterations in translation and abundance together with instances of translational offsetting. To validate the observed changes in gene expression, we selected 200 genes whose translation or abundance was increased or decreased in *TSC1*^−/−^ as compared to *TSC1*^+/+^ NPCs and quantified their expression pattern using the NanoString nCounter Gene Expression Analysis technology. This largely confirmed the gene expression modes (Fig. 1D). To further validate changes in mRNA translation, we focused on a subset of genes that did or did not show accompanying alterations in mRNA levels (Fig. 1E, left), and performed RT-qPCR using total or polysome-associated mRNA as input. This identified larger TSC1-associated changes in polysome-associated mRNA as compared to total mRNA for all genes in the validation subset (Fig. 1E, right). Next, we assessed the potential functional impact of these changes in translational efficiencies using ClueGO-based gene ontology analysis [56]. This revealed that translation of mRNAs encoding proteins annotated to, e.g., ion transport, immune system function and RNA polymerase II were selectively altered (Fig. 1F and Additional file 4: Table S3A-B). Overall, these data show that loss of TSC1 function in NPCs leads to reprogrammed gene expression via alterations in both mRNA abundance and translation.

### Alterations of mRNA translation in ASD patient compared with control brains

**Fig. 2:**
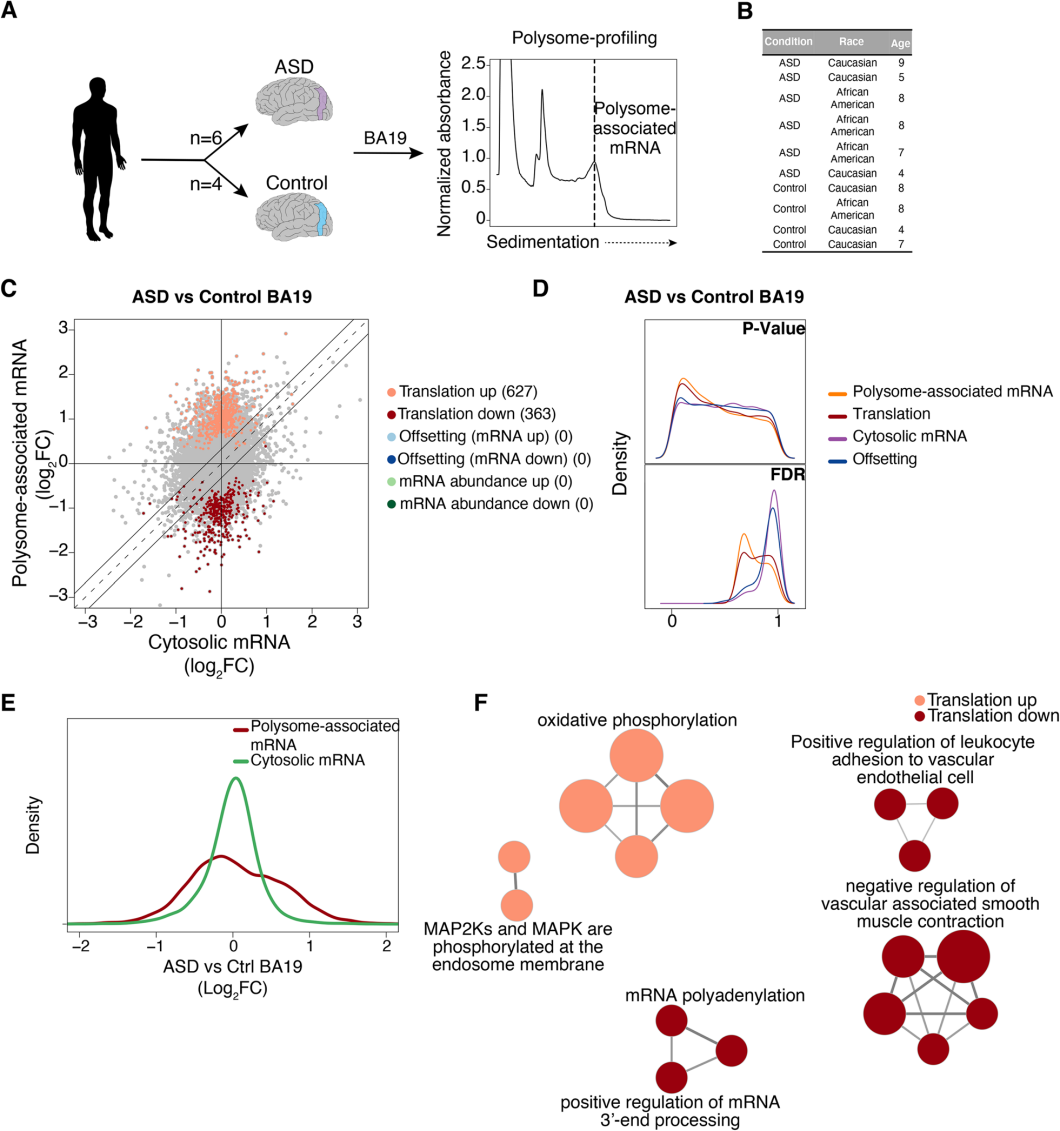
ASD-associated alterations of mRNA translation in BA19. **A** Overview of experimental setup for polysome profiling of BA19 brain samples. Polysome-tracings were obtained using the optimized sucrose gradient as described in “Methods” section. **B** Characteristics of ASD-affected donors and controls included in the study. **C** Scatter plot comparing polysome-associated to cytosolic mRNA log_2_ fold changes for the ASD versus Control BA19 (similar to Fig. 1B). **D** Kernel densities of p values or FDRs (from anota2seq analysis) for the comparison of ASD versus Control BA19 (similar to Fig. 1C). **E** Kernel densities of log_2_ fold changes for total cytosolic and polysome-associated mRNA comparing ASD versus Control BA19. **F** Gene ontology analysis (similar to Fig. 1F) for the comparison of ASD to Control BA19

We next sought to assess whether similar patterns of mRNA translation to those identified as TSC1-associated in NPCs are observed in postmortem brain tissue from ASD patients. To this end, we obtained 10 brain samples collected from neurotypical and ASD-affected male donors matched for age (4–9 years old). Furthermore, all samples originated from the Brodmann Area 19 (BA19), a part of the occipital lobe cortex involved in responses to visual stimuli (Fig. 2A, B). As donors are incompletely annotated for comorbidities, medications and severity, these factors were not considered in downstream analyses. As brain samples were small, we used a recently developed optimized polysome profiling technique amenable to small tissue samples [50] to obtain, to our knowledge, a first dataset of transcriptome-wide alterations of mRNA translation in ASD-affected postmortem brain tissue. To know whether variations in coding sequence in TSC1 or TSC2 were seen in ASD donor samples, RNA sequencing data were analyzed employing the Genome Analysis Toolkit (GATK, https://gatk.broadinstitute.org/hc/en-us), which demonstrated no coding sequence variations in *TSC1* and two independent benign/synonymous *TSC2* variations, one in each of two ASD samples (not shown). Like above (Fig. 1B, C), we analyzed the resulting dataset using anota2seq [54, 55] (Fig. 2C, D). Despite the limited statistical power to detect gene expression changes due to the scarce availability of matched ASD and control samples, we observed an enrichment of low p values for ASD-associated alterations in translation (Fig. 2D). This was further supported by a left-shifted distribution of FDRs (Fig. 2D) together with a larger range of fold changes (ASD versus control) for polysome-associated mRNA as compared to total cytosolic mRNA (Fig. 2E). Strikingly, ClueGO gene ontology analysis revealed that genes regulated via translation are involved in previously ASD-implicated functions including oxidative phosphorylation, MAPK pathway and alternative polyadenylation (Fig. 2F and Additional file 4: Table S3C-D). Therefore, albeit limited by the low availability of tissues for studies, these data support reprogrammed translation in postmortem brain tissue from donors affected by ASD.

### ***TSC1***^***−/−***^ NPCs recapitulate mRNA translation in brains of ASD patients

**Fig. 3:**
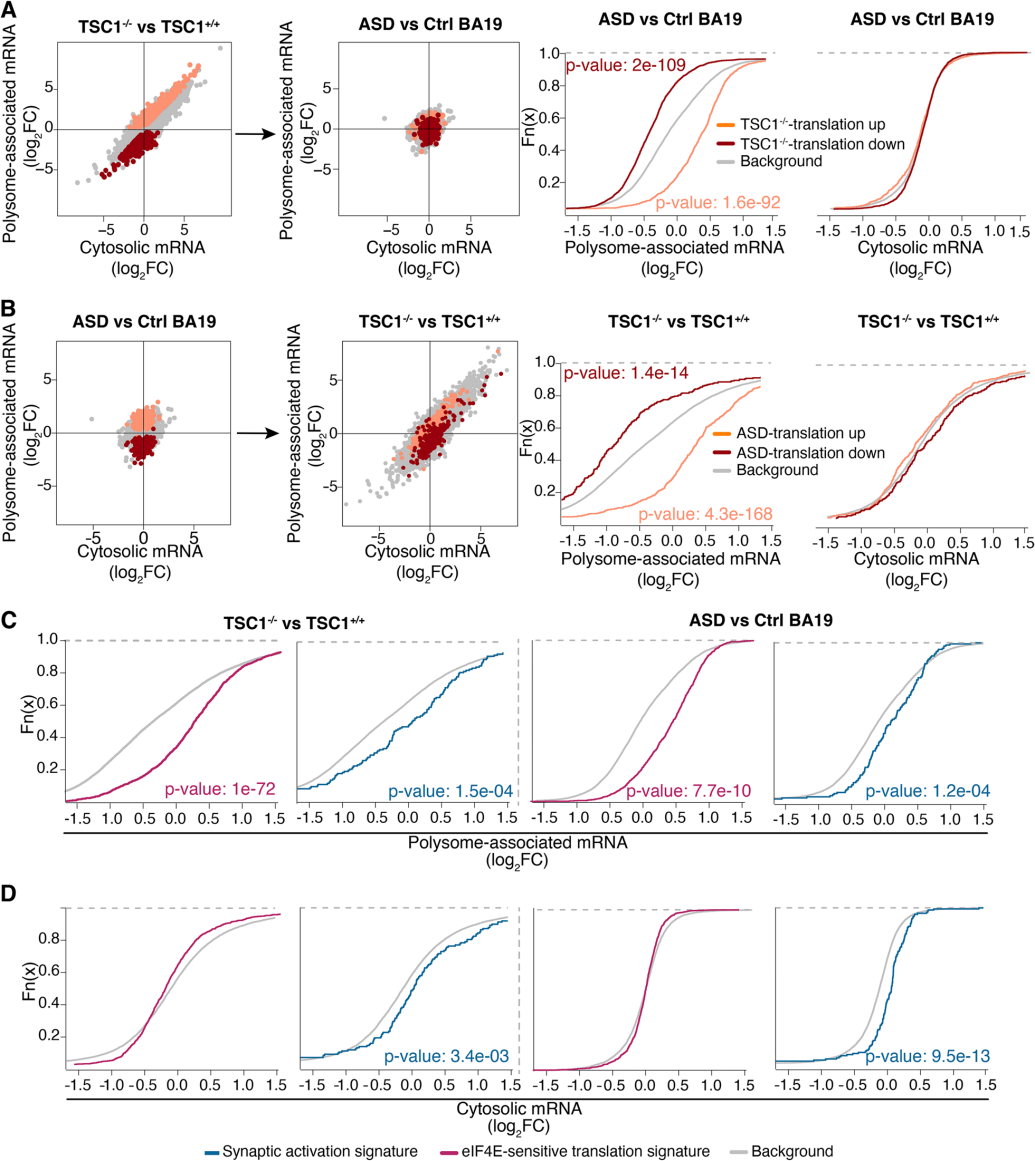
*TSC1*^−/−^ NPCs recapitulate translation observed in ASD BA19 samples. **A** Scatter plots from anota2seq analysis (left two panels) where transcripts whose translation was activated or suppressed in *TSC1*^−/−^ versus *TSC1*^+/+^ NPCs (i.e., from Fig. 1B) are indicated in *TSC1*^−/−^ versus *TSC1*^+/+^ NPCs and ASD versus control comparisons. Shown are also empirical cumulative distribution function (ECDF) plots assessing regulation of the same gene sets relative to the background (i.e., genes not in gene set) for polysome-associated and cytosolic mRNA log_2_ fold changes (ASD vs control BA19; two rightmost plots). Wilcoxon rank-sum test p values are indicated for the comparison of each gene set relative to the background. **B** Scatter plots and ECDF plots (similar to **A**) assessing regulation of transcripts whose translation were altered in ASD versus control BA19 in the comparison of *TSC1*^−/−^ versus *TSC1*^+/+^ NPCs. **C, D** ECDF plots assessing genes related to synaptic activation and transcripts whose translation increased upon eIF4E overexpression. Signatures were evaluated in *TSC1*^−/−^ versus *TSC1*^+/+^ NPCs (left two panels) and ASD versus control BA19 (right two panels). Fold changes were calculated using polysome-associated mRNA (**C**) or cytosolic mRNA (**D**). Wilcoxon rank-sum test p values are indicated for the comparison of each gene set relative to the background (i.e., genes not in gene sets)

We compared the datasets obtained from NPCs and BA19 samples to determine if there are overlaps in gene expression programs. First, we assessed whether transcripts with TSC1-sensitive translation in NPCs showed altered gene expression when comparing samples originating from ASD versus control brains. Strikingly, transcripts whose translation was increased when comparing *TSC1*^−/−^ to *TSC1*^+/+^ NPCs (i.e., those identified in Fig. 1B) showed increased translation also when comparing ASD to controls (as levels of polysome-associated mRNA were increased while total mRNA levels where unchanged; Fig. 3A). Similarly, transcripts that were translationally suppressed in *TSC1*^*−*/−^ relative to *TSC1*^+/+^ NPCs were also translationally suppressed in BA19 from ASD relative to control subjects (Fig. 3A). We next performed the reciprocal analysis by assessing whether transcripts with altered translation in ASD versus control samples (i.e., those identified in Fig. 3A) also showed TSC1-sensitive expression in NPCs. Indeed, transcripts showing increased or decreased translation when comparing BA19 samples from ASD to controls revealed similar translation patterns when comparing *TSC1*^−/−^ to *TSC1*^+/+^ NPCs (Fig. 3B). Accordingly, the NPC model captures alterations in mRNA translation occurring in brains of ASD patients.

To further explore similarities between the two datasets, we assessed whether synaptic genes (Additional file 5: Table S4A) [58], and transcripts whose translation was previously identified as induced upon overexpression of eIF4E (“eIF4E-sensitive”) [57], are regulated in the NPC and ASD datasets. In agreement with hyperactivation of the mTORC1-eIF4E axis, previously identified eIF4E-sensitive transcripts were translationally activated in *TSC1*^*−*/−^ relative to *TSC1*^+/+^ NPCs as well as in ASD versus control brains (as judged by their increased polysome-association in the absence of changes in total mRNA; Fig. 3C, D and Additional file 5: Table S4B). In contrast, synaptic genes showed increased levels of both polysome-associated and cytosolic mRNA in both datasets (Fig. 3C, D). Furthermore, ClueGO gene ontology analysis highlighted an overlap of functions enriched among proteins encoded by transcripts with altered translation in *TSC1*^*−*/−^ and ASD relative to their controls (Additional file 1: Fig. S4A–B and Additional file 4: Table S3E–F). This included oxidative phosphorylation and cation transport as activated, while transcription-related processes were suppressed. These findings further underline that a more complete understanding of ASD-associated gene expression changes and their mechanistic underpinnings requires studies of mRNA translation.

### Rapamycin only partially reverses TSC1-associated translation

**Fig. 4:**
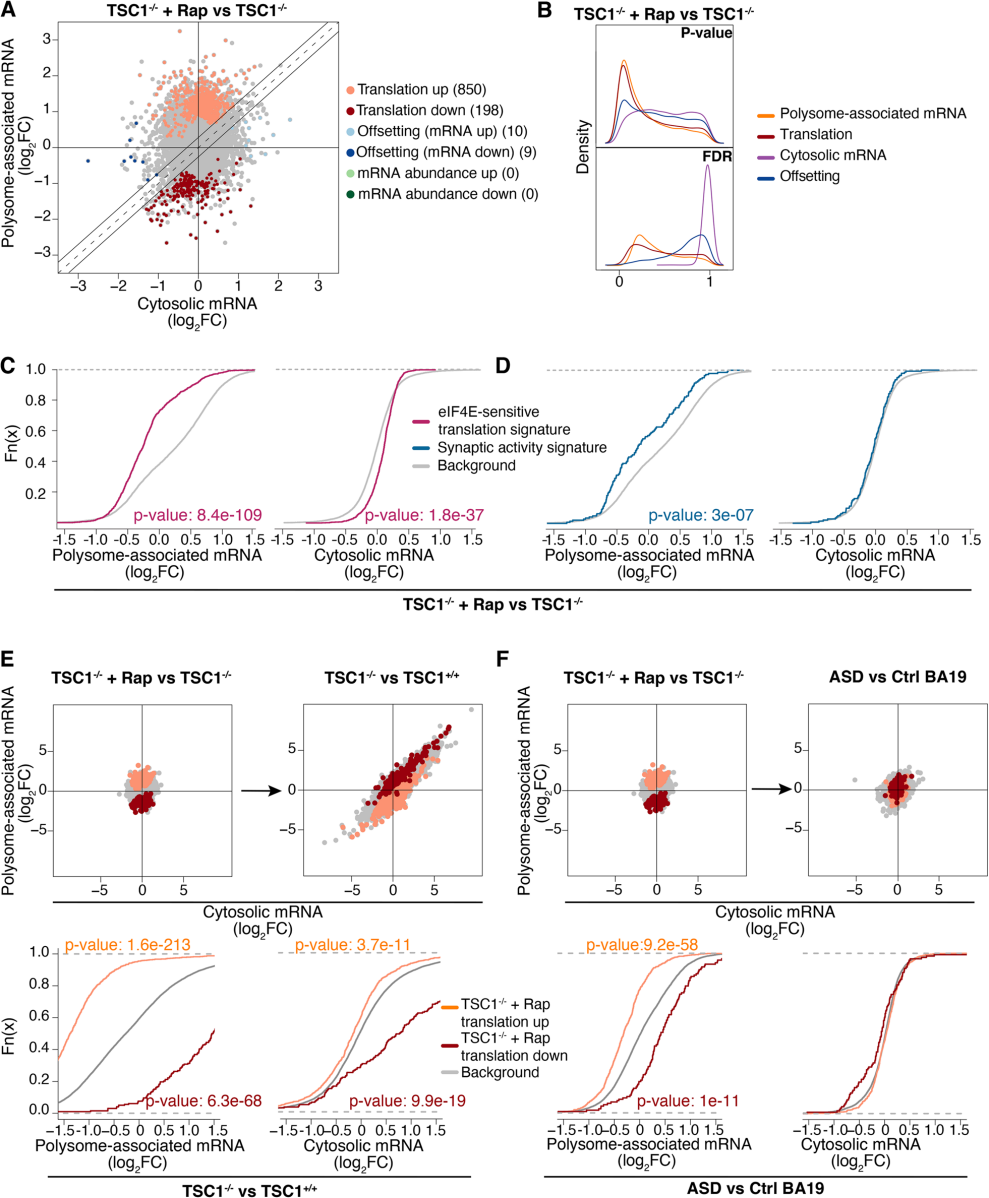
Rapamycin partially reverses TSC1- and ASD-associated translation. **A**, **B** anota2seq analysis of *TSC1*^−/−^ cells in the presence versus absence of rapamycin (similar to Fig. 1B, C). **C, D** ECDF plots for rapamycin sensitivity of eIF4E and synaptic activity signatures in *TSC1*^−/−^ cells (similar to Fig. 3C, D). **E, F** Scatter plots (similar to Fig. 3A) assessing how transcripts showing rapamycin-sensitive translation are modulated in the *TSC1*^−/−^ versus *TSC1*^+/+^ NPCs (**E**) or ASD versus control BA19 (**F**) comparisons

As discussed above, *TSC1* loss leads to hyperactivated mTORC1 signaling, which affects mRNA translation both globally and selectively [31, 40]. Accordingly, mTORC1 inhibitors have been considered as a strategy to treat phenotypes resulting from loss of TSC1 [5, 6, 8]. To assess whether these agents reverse TSC1-associated mRNA translation in NPCs, we used polysome profiling in cells treated with the mTORC1 inhibitor rapamycin (Fig. 1A). Anota2seq analysis comparing *TSC1*^*−*/−^ NPCs in the presence or absence of rapamycin revealed that short-term treatment almost exclusively modulated mRNA translation (Fig. 4A, B). Consistent with rapamycin inhibiting translation via the mTORC1/eIF4E axis, translation of mRNAs previously identified as eIF4E-sensitive (same subset as in Fig. 3C, D) was suppressed in rapamycin-treated *TSC1*^−/−^ NPCs (Fig. 4C and Additional file 5: Table S4B). In addition, rapamycin reduced polysome-association of mRNAs transcribed from a small set of genes with synaptic activity (Fig. 4D and Additional file 5: Table S4A; same subset as in Fig. 3C, D). Next, using the same strategy as above (Fig. 3A, B), we assessed whether identified rapamycin-sensitive translation is modulated when comparing *TSC1*^*−*/−^ versus *TSC1*^+/+^ NPCs or ASD versus control brains. Consistent with hyperactivation of eIF4E-sensitive translation downstream of mTORC1 (Fig. 3C, D), transcripts with suppressed translation upon rapamycin treatment showed hyperactive translation when contrasting *TSC1*^*−*/−^ to *TSC1*^+/+^ NPCs or ASD versus control brains (Fig. 4E, F).

**Fig. 5:**
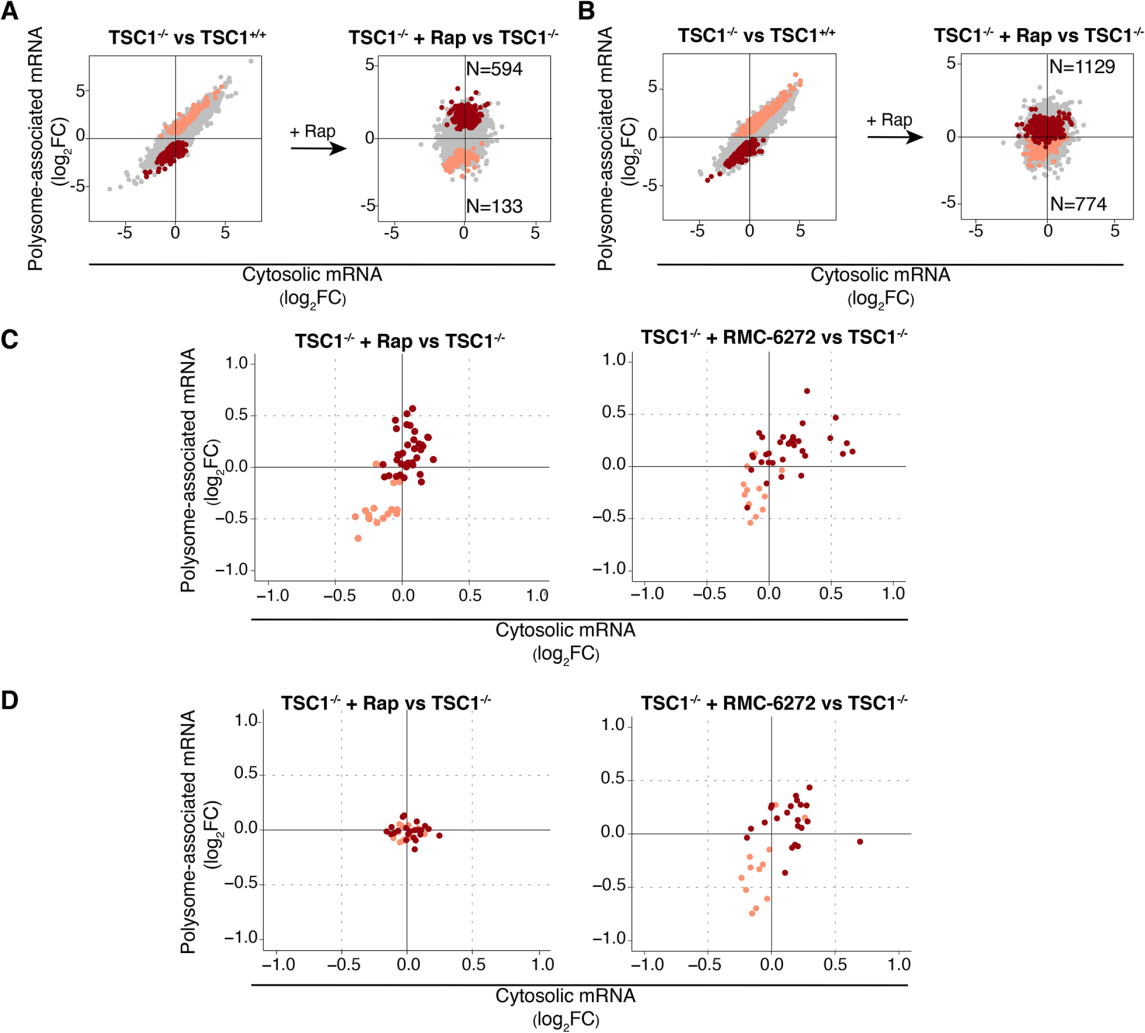
RMC-6272 reverses rapamycin-insensitive TSC1-associated alterations in mRNA translation. **A, B** Scatter plots (similar to Fig. 3A) of transcripts showing TSC1-associated mRNA translation that were reversed (**A**) or insensitive (**B**) to rapamycin treatment. The number of transcripts following each pattern of regulation is indicated. **C, D** Scatter plots of log2 fold changes from NanoString nCounter assays for transcripts showing TSC1-associated mRNA translation that was sensitive (**C**, left) or insensitive (**D**, left) to rapamycin (according to RNA sequencing-based quantification) but sensitive to RMC-6272 (**C**, **D**, right). Fold changes were calculated between cells treated with rapamycin (left) or RMC-6272 (right) relative to control (DMSO)

Although rapamycin reverses TSC1-associated changes of mRNA translation in NPCs (Fig. 4E), previous studies have indicated that the effect on the translatome is only partial—likely due to rapamycin incompletely reducing phosphorylation of 4E-BPs [65]. Consistent with incomplete reversal of mTORC1-sensitive translation by rapamycin, neurite outgrowth in *TSC1*^−/−^ cells was not rescued by rapamycin [24]. To assess the extent to which TSC1-associated translation is sensitive to rapamycin, we separated mRNAs whose translation was altered in *TSC1*^−/−^ versus *TSC1*^+/+^ NPCs into subsets with rapamycin-reversed (Fig. 5A) or -insensitive translation (Fig. 5B). Indeed, this revealed that only a subset of TSC1-associated translation changes was reversed by rapamycin, while most transcripts were insensitive (Additional file 6: Table S5). Consistently, anota2seq analysis comparing polysome profiling data from rapamycin-treated *TSC1*^*−/−*^ to non-treated *TSC1*^+*/*+^NPCs indicated ample differences in gene expression (Additional file 1: Fig. S5A–B). A new generation of bi-steric mTORC1-selective inhibitors, which suppresses phosphorylation of 4E-BP1 to a greater extent, has recently been developed [17, 18]. To evaluate whether these inhibitors may reverse TSC1-associated mRNA translation more efficiently than rapamycin, we used our NanoString nCounter Gene Expression Analysis code set to analyze effects on translation in *TSC1*^−/−^ NPCs treated with RMC-6272, an mTORC1-selective bi-steric third-generation inhibitor [18, 66] compared to rapamycin. We focused the analysis on NanoString targets with TSC1-associated changes in mRNA translation, separated these into those whose translation was reversed or insensitive to rapamycin, and compared the effects of mTOR allosteric inhibition with mTORC1 bi-steric inhibition. As expected, transcripts showing rapamycin-sensitive translation were also sensitive to RMC-6272 (Fig. 5C). Conversely, transcripts whose translation was insensitive to rapamycin (Fig. 5D, left) were largely sensitive to RMC-6272 (Fig. 5D, right). Importantly, a subset of differentially translated transcripts emerged from our NanoString data that remained rapamycin-insensitive but were reversed by RMC-6272, that were previously associated with ASD, NDD or synaptic activity (Additional file 7: Table S6) [67, 68]. Accordingly, these studies suggest that more efficient inhibition of mTORC1 with a bi-steric inhibitor reverses TSC1-associated alterations in mRNA translation to a greater extent than rapamycin and may therefore have distinct effects on ASD-associated phenotypes.

### TSC1 NPCs show genotype-dependent phenotypes that are reversed by RMC-6272

**Fig. 6:**
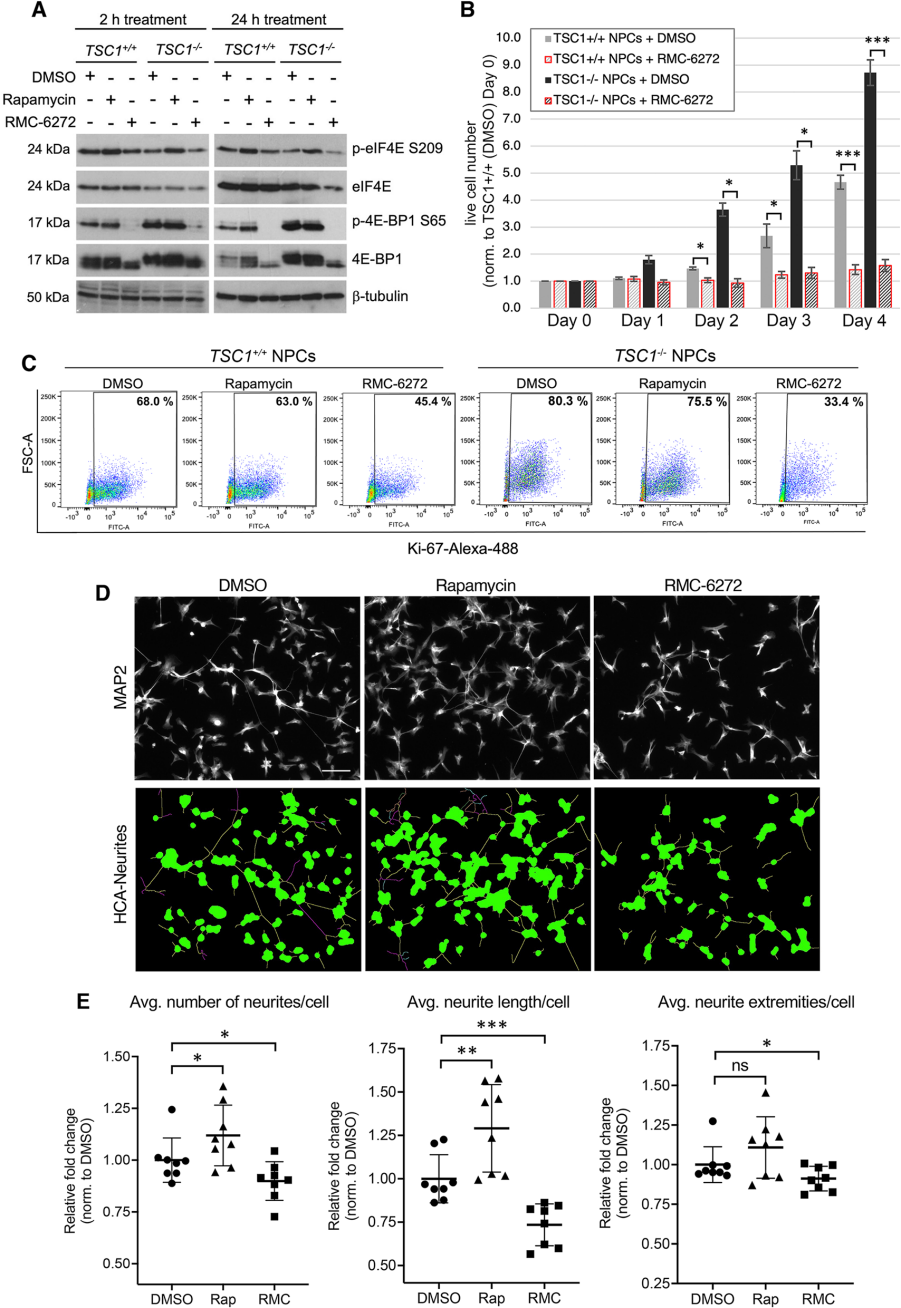
RMC-6272 rescues early neurodevelopmental phenotypes in *TSC1*^−/−^ NPCs. **A** Immunoblotting for indicated proteins in *TSC1*^+*/*+^ and *TSC1*^*−/−*^ NPCs treated with 10 nM RMC-6272 or 50 nM rapamycin (2 or 24 h, *n* = 3). β-tubulin serves as a loading control. Images have been cropped for clarity and conciseness and entire blots are shown in Additional file 9. **B** Proliferation rate for NPCs after treatment with DMSO or 10 nM RMC-6272 was quantified at Day 0 (D0, cell number at seeding), and live cell numbers were assessed at D1-4 using trypan blue exclusion. Data represent average fold change normalized to *TSC1*^+*/*+^ NPCs at D0 (n = 3 ± SD). **C** Representative flow cytometry plots indicate Ki-67-positive populations upon 72 h treatment with DMSO, 50 nM rapamycin or 10 nM RMC-6272 for *TSC1*^+*/*+^ NPCs (left) and *TSC1*^*−/−*^ NPCs (right). Graphs were generated by plotting forward scatter-area (FSC-A) versus FITC-area (representing cells positive for Ki-67-Alexa488). Boxed regions represent gating of Ki-67 positively stained cells with percentages shown. **D** Representative images are shown for immunofluorescence staining of *TSC1*^*−/−*^ NPCs treated with DMSO (left), 50 nM rapamycin (middle) or 10 nM RMC-6272 (right) using the neuronal marker microtubule associated protein 2 (MAP2, grayscale, top) as well as traced images generated employing HCA-Vision image quantitation software (bottom), which traces the cell body along branching layers of the neurite arborization. Scale bar = 100 µm. **E** Quantitation of neurite number (left), length (middle) and extremities (right) in *TSC1*^*−/−*^ NPCs treated with 10 nM RMC-6272 (RMC) or 50 nM rapamycin (Rap) for 48 h are shown. Data represent analysis of 8 non-overlapping field images/treatment generated using GraphPad Prism9 with relative fold change normalized to DMSO-treated NPCs (mean, ± SD). *p < 0.05, **p < 0.01, ***p < 0.001, ns not significant, p values were calculated using Student’s t test (**B**, **E**)

We have previously shown that *TSC1*^*−/−*^ NPCs display a significant increase in cell size, neurite number and length, as well as proliferation compared to *TSC1*^+*/*+^ NPCs [24]. Immunoblotting of *TSC1*^+*/*+^ and *TSC1*^*−/−*^ NPCs treated with rapamycin or RMC-6272 for 2 or 24 h showed attenuation of phosphorylated ribosomal S6 (p-S6 S240/244) but only RMC-6272 reversed phosphorylation of 4E-BP1. In addition, consistent with our previous report [24], p-eIF4E was increased upon rapamycin treatment, but remained unchanged in RMC-6272 treated cells (Fig. 6A and Additional file 1: Fig. S3B). Increased cell size is a hallmark of mTORC1 hyperactivation and, consistently, treatment of NPCs with either 50 nM rapamycin or 10 nM RMC-6272 for 24 h led to a similar reduction in cell size when compared with DMSO-treated control cells (Additional file 1: Fig. S6A). For NPC proliferation, unlike rapamycin that showed no effect in our previous report [24], RMC-6272 significantly rescued the increased proliferation. Treatment with 10 nM RMC-6272, which started at Day 1 (D1) after seeding, inhibited proliferation of *TSC1*^+*/*+^ and *TSC1*^*−/−*^ NPCs, with cell numbers through D4 remaining close to seeding (D0; Fig. 6B). In addition, flow-cytometry-based Ki-67 analysis confirmed increased proliferation in DMSO-treated *TSC1*^−/−^ NPCs compared with *TSC1*^+*/*+^ NPCs. Treatment of *TSC1*^+*/*+^ and *TSC1*^−/−^ NPCs with 10 nM RMC-6272 revealed decreased Ki-67, which was superior to 50 nM rapamycin (Fig. 6C). As a further validation, in real-time cellular imaging-based assays carried out over 48 h, *TSC1*^*−/−*^ showed significant increase in proliferation versus *TSC1*^+*/*+^ NPCs. Treatment with 10 nM RMC-6272 significantly lowered proliferation of *TSC1*^*−/−*^ NPCs, reducing nuclei number close to *TSC1*^+*/*+^ levels (Additional file 1: Fig. S6B).

Neurite analysis was carried out after 48 h of treatment using HCA-Vision image analysis software to examine three parameters: (1) average number of neurites/cell, (2) overall neurite outgrowth—including average length of all neurite segments/cell and (3) average number of extremities/cell—comprising terminal endpoints of all primary and branched outgrowths [62]. Strikingly, RMC-6272 in *TSC1*^*−/−*^ NPCs led to a significant reduction in all three parameters of neurite number, length and extremities compared with DMSO-treated cells. Conversely, rapamycin treatment showed no decrease in neurite number, length or extremities, and in fact showed an increase in number and length (Fig. 6D, E). It should also be noted that NPCs derived from the parental patient cell line bearing the heterozygous *TSC1* exon 15 mutation [24] also demonstrated a significant reduction in all three parameters upon treatment with RMC-6272, with no decrease upon rapamycin treatment. In CRISPR-corrected WT NPCs, RMC-6272 also reduced all three parameters, whereas rapamycin failed to reduce the number and extremities of neurites (Additional file 1: Fig. S7). Taken together, these data support that treatment with the third-generation mTORC1 inhibitor leads to more complete inhibition of mTORC1 downstream targets including 4E-BP1 and is more potent than rapamycin in rescuing the altered early neurodevelopmental phenotypes such as neurite outgrowth and proliferation in *TSC1*-mutant NPCs.

## Discussion

It is well established that loss of TSC1 or TSC2 results in activation of mTORC1 signaling, which has led to FDA approval for treatment of TSC-associated tumors with first-generation mTORC1 inhibitors such as everolimus/RAD-001. However, rapalogs have not been very effective for treating TSC-associated neuropsychiatric defects and autism [10, 11]. The mTORC1 signaling pathway plays a critical role in protein synthesis in normal cells including stem cells, and in human disease through regulation of translation initiation (reviewed in [69, 70]).The mTORC1/eIF4F axis is therefore critical in shaping the proteome. Although transcriptome-wide studies of TSC-associated mRNA translation have been performed in mouse embryonic fibroblasts [71], the effects of TSC-loss in patient-derived NPCs have not been assessed. Here, we sought to bridge this gap in knowledge.

Here, we, for the first time, reveal the complex pattern of gene expression alterations downstream of TSC1 loss in patient-derived NPCs encompassing both changes in mRNA abundance as well as numerous alterations in translational efficiencies. Interestingly, TSC1-dependent alterations in mRNA translation observed in NPCs were largely recapitulated in human ASD brains. In addition, our study of TSC1-associated gene expression also indicated ample translational offsetting, which denotes a poorly characterized gene expression mode possibly representing adaptation [63, 64]. Although this may be of interest to fully understand how TSC1 loss reprograms gene expression, as this mode of regulation was not observed in human ASD brains, we did not study it further herein. Furthermore, although polysome profiling revealed a partial reversal of TSC1-associated gene expression alterations following rapamycin treatment, most genes related to neural activity/synaptic regulation or ASD that showed TSC1-dependent translation were rapamycin-insensitive. Bi-steric mTORC1-selective inhibitors, including RMC-6272 and its clinical counterpart RMC-5552, show strong anti-tumor activity either alone or when combined with other treatments in several preclinical cancer models. RMC-5552 also demonstrates preliminary evidence of anti-tumor activity at tolerated doses [18, 66]. Here, we reveal that RMC-6272 is not only more potent than rapamycin in inhibiting mTORC1, but also reverses some of the translational changes not reversed by rapamycin (Fig. 5D). These findings are consistent with previous comparisons between the effects of rapamycin and the active site mTOR inhibitor PP242 on transcriptome-wide translation in cancer cells [65]. More importantly, unlike rapamycin, RMC-6272 can rescue early neurodevelopmental phenotypes such as proliferation and neurite outgrowth in *TSC1*^*−/−*^ NPCs (Fig. 6 and [24]), raising the question whether 4E-BP1-dependent translation could be essential for some of the neurodevelopmental phenotypes in TSC and other mTORC1-activated neurodevelopmental disorders.

In addition to TSC, dysregulated mTORC1 signaling is also observed in other syndromic ASDs such as Cowden syndrome/PTEN hamartoma syndrome, Fragile X syndrome, RASopathies including NF1, Angelman syndrome and Rett syndrome, as well as idiopathic ASD [72, 73], raising the possibility that cap-dependent translation downstream of mTORC1 could play an essential role in neurodevelopmental and neuropsychiatric disorders. Many of the recent large-scale studies have focused on the transcriptome for understanding gene expression changes in the pathophysiology of ASD and other neuropsychiatric disorders [68, 74, 75]. Defining changes in mRNA translation in neurodevelopmental and neuropsychiatric disorders remains largely unexplored and our study here describing such changes in TSC1 patient-derived neural progenitor cells will likely open avenues for correlating transcriptional alterations with changes in mRNA translation in ASD and other neurodevelopmental disorders with dysregulated mTORC1 signaling.

## Limitations

The presented study relies on a single pair of NPCs with null or WT *TSC1*. While this study design allows for a deeper focus on the role of TSC1 in modulating mRNA translation in NPCs, future studies will benefit from assessing translatomes in additional isogenic NPC sets from TSC patients also harboring TSC2 mutations. Moreover, due to a low availability of postmortem specimens, the size of the BD19 study was small. Although this design still allowed for a pioneering assessment of mRNA translation in brains of ASD patients, it reduced the statistical power to detect gene expression changes and eliminated the possibility of identifying potential subsets of patients with distinct translation patterns. Moreover, as the size of BA19 samples was small, alterations in mRNA translation could not be validated using, e.g., NanoString methodology. To more completely understand the spectrum of mRNA translation alterations in ASD, a larger sample size is required. For statistical significance in Fig. 6, in addition to a data threshold of p < 0.01 and p < 0.001, some data showing modest, yet reproducible effects (p < 0.05) are also presented and indicated in the figure legend.

## Conclusions

Our approach employing polysome profiling of TSC1 patient-derived isogenic NPCs revealed numerous changes in mRNA levels and translation associated with *TSC1*-loss. Many of these changes in mRNA translation were recapitulated in human ASD brain samples. Treatment of NPCs using rapamycin, the only FDA approved treatment for TSC patients to date, led to a partial reversal in TSC1-associated translation, yet most genes related to neural activity/synaptic regulation or ASD remained unchanged. Conversely, the third-generation bi-steric mTORC1-selective inhibitor RMC-6272 not only reversed many of these rapamycin-insensitive genes, but also rescued the rapamycin-insensitive early neurodevelopmental phenotypes observed in *TSC1-/-* NPCs including neurite outgrowth and proliferation. These findings suggest that more efficient targeting of the mTORC1-eIF4E axis may prove to be a superior treatment strategy for TAND.

### Supplementary Information

**Additional file 1**. **Figure S1.** Quality control of RNA sequencing data of cytosolic and polysome-associated mRNA isolated from NPCs of different conditions. **A** Barplot showing overall number of reads that are aligned to adapter sequences, rRNA sequences, assigned to genes as well as unmapped/unassigned reads. **B** Boxplot showing TMM-log2 normalized counts of *TSC1* transcript in *TSC1*^*−/−*^ (red) and *TSC1*^+*/*+^ (blue) NPCs. **C** Scree-plot showing the percentage of variance explained by PC1-PC10.** D** Correlation of principal components (PC1-PC9) to experimental factors.** E** Projection of samples in principal components 1 and 2, with samples shaped according to library type (circle: polysome-associated mRNA; triangle: cytosolic mRNA) and colored according to genotype of samples (*TSC1*^*−/−*^, red; *TSC1*^+*/*+^, blue). **Figure S2.** Quality control of RNA sequencing data of postmortem samples of BA19 ASD and control.** A** Barplot showing overall number of reads that are aligned to adapter sequences, assigned to genes as well as unmapped/unassigned reads. **B** Scree-plot showing the percentage of variance explained by PC1-PC10. **C** Correlation of principal components (PC1-PC9) to experimental factors. **D** Projection of samples in principal components 1 and 2, with samples shaped according to library type (circle: polysome-associated mRNA; triangle:cytosolic mRNA) and colored according to condition (ASD: red; Control: blue). **Figure S3.** Immunoblotting in NPCs. **A** Immunoblotting for TSC1 in *TSC1*^−/−^ compared with CRISPR-corrected *TSC1*^+*/*+^ NPCs. Ribosomal S6 protein serves as a loading control. **B** Immunoblot of NPCs treated with rapamycin (50 nM) and RMC-6272 (10 nM) for indicated proteins. β-tubulin served as a loading control. Images have been cropped for clarity and conciseness, and entire blots are shown in Additional file 9. **Figure S4**. Gene ontology analysis comparison for ASD and *TSC1*^*−/−*^ NPCs. **A and B** Gene ontology analysis (similar to Fig. 1F) for genes categorized as “translation up” in *TSC1*^*−/−*^ versus *TSC1*^+*/*+^ and ASD versus Ctrl BA19 comparisons (A); and genes categorized as “translation down” in *TSC1*^*−/−*^ and *TSC1*^+*/*+^ and ASD versus Ctrl BA19 comparisons (B). The analysis was performed using ClueGO in “cluster mode.” **Figure S5.** Comparison of rapamycin-treated *TSC1*^*−/−*^ to non-treated *TSC1*^+*/*+^ NPCs. **A and B** anota2seq analysis (A) and kernel densities for p value or FDR from anota2seq analysis (B) are shown comparing rapamycin-treated *TSC1*^−/−^ to non-treated *TSC1*^+*/*+^ NPCs. (similar to Fig. 1B-C). **Figure S6.** Additional data related to changes in cells size and proliferation. **A** Bright field images (left panel) and cell size quantitation (right panel) of *TSC1*^+*/*+^ and *TSC1*^*−/−*^ NPCs treated with 50 nM rapamycin or 10 nM RMC-6272 (n = 3 ± SD) are shown. Scale bar = 100 µm. **B** *TSC1*^+*/*+^ and *TSC1*^*−/−*^ NPCs were treated with 10 nM RMC-6272 or DMSO as a control along with 1:500 dilution of the fluorescent nuclear marker NucSpot650 to visualize cell nuclei in the near infrared (NIR) spectrum. Using the Incucyte SX5 system, images were taken every 2 h for a total of 48 h. Graphs of NIR mean intensity (NIRCU) were generated using GraphPad Prism9 showing the average nuclei number/image field (36 non-overlapping image fields/well). Data represent three biological replicates per treatment group (± SEM). *p < 0.05, **p < 0.01, ***p < 0.001 calculated by Student’s t test. **Figure S7.** Additional data related to changes in neurite outgrowth. **A and B** Quantitation of neurite number (left), length (middle) and extremities (right) from trace images are shown for immunofluorescence staining of *TSC1*^±^ (A) and *TSC1*^+*/*+^ (B) NPCs treated with DMSO, 50 nM rapamycin or 10 nM RMC-6272 using the neuronal marker MAP2 and HCA-Vision image quantitation software. Data represent eight non-overlapping field images/treatment generated using GraphPad Prism9 with relative fold change normalized to DMSO-treated NPCs (mean, ± SD). ** p < 0.01, ***p < 0.001, ****p < 0.0001, ns = not significant calculated by Student’s t test (**A**-**B**).

**Additional file 2**. Primary antibodies used in this study.

**Additional file 3**. Human RT-qPCR primers used in this study.

**Additional file 4**. ClueGO-based gene ontology analysis.

**Additional file 5**. Gene expression data for genes in synaptic- and eIF4E-signatures.

**Additional file 6**. Differentially translated genes in *TSC1*^*−/−*^ as compared to *TSC1*^+*/*+^ NPCs that are insensitive or sensitive to rapamycin treatment.

**Additional file 7**. Differentially translated genes in *TSC1*^*−/−*^ as compared to *TSC1*^+*/*+^ NPCs related to ASD, NDD and synaptic activity that are sensitive to RMC-6272 but not rapamycin treatment.

**Additional file 8**. Details related to each figure regarding iPSC-derived NPCs and number of independent experiments and replicates.

**Additional file 9**. Additional figures showing full length blots for Fig. 6A and Fig. S3.

## Data Availability

The polysome-profiling datasets supporting the conclusions of this article are included within the article (and its additional file(s)), and the full datasets are deposited into Gene Expression Omnibus (GEO) with accession numbers GSE239412 and GSE236761.

## Abbreviations

4E-BPs: EIF4E-binding proteins
APV: Analysis of partial variance
ASD: Autism spectrum disorder
BA19: Brodmann Area 19 (BA19) brain region, a part of the occipital lobe cortex involved in responses to visual stimuli
BH: Benjamini–Hochberg adjustment of p value
CHX: Cycloheximide
CRISPR: Clustered regularly interspaced short palindromic repeats
ECDFs: Empirical cumulative distribution functions
eIF4E: Eukaryotic translation initiation factor 4E
FDR: False discovery rate
GO: Gene ontology
ID: Intellectual disability
iPSC: Induced pluripotent stem cells
LOG2FC: Log2 fold change
MAP2: Microtubule-associated protein 2
mTORC1: Mammalian/mechanistic target of rapamycin complex 1
ND: Neurodevelopment/neurodevelopmental
NDD: Neurodevelopmental disorders
NPC: Neural progenitor cells
PCA: Principal component analysis
PFA: Paraformaldehyde
Rap/rapalogs: Rapamycin/rapamycin-analogs
RNA-seq: RNA sequencing
RT-qPCR: Reverse transcriptase quantitative PCR
S6: Ribosomal protein S6
TAND: TSC-associated neuropsychiatric disorders
TSC: Tuberous sclerosis complex
vsn: Variance stabilizing normalization
