# Supplementary material for: Translatome analysis of tuberous sclerosis complex 1 patient-derived neural progenitor cells reveals rapamycin-dependent and independent alterations
Source: Mol Autism. 2023 Oct 25;14:39. doi: 10.1186/s13229-023-00572-3 (PMC10601155; doi:10.1186/s13229-023-00572-3)

Additional File 1

Additional Figure S1

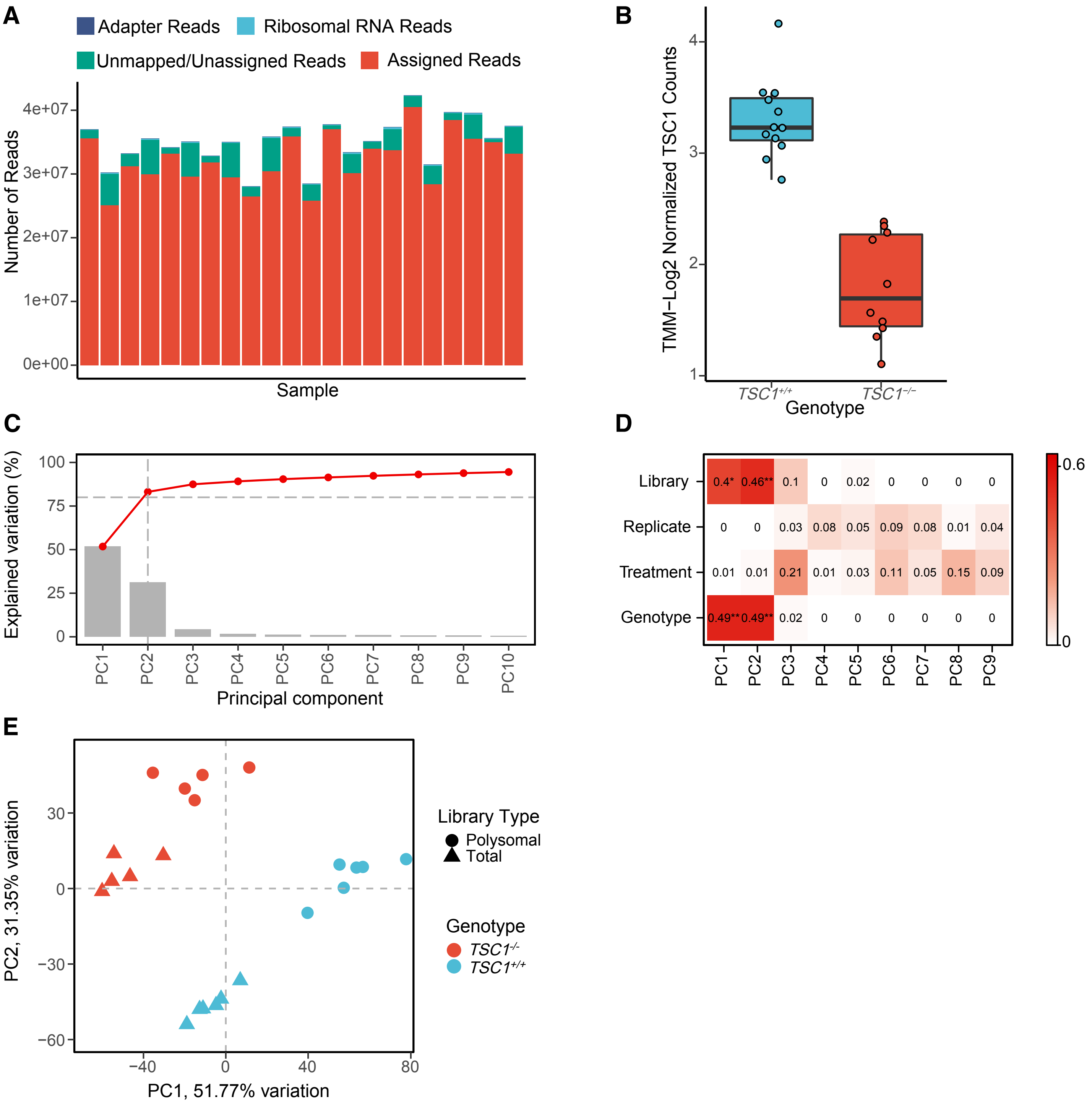

Additional Figure S2

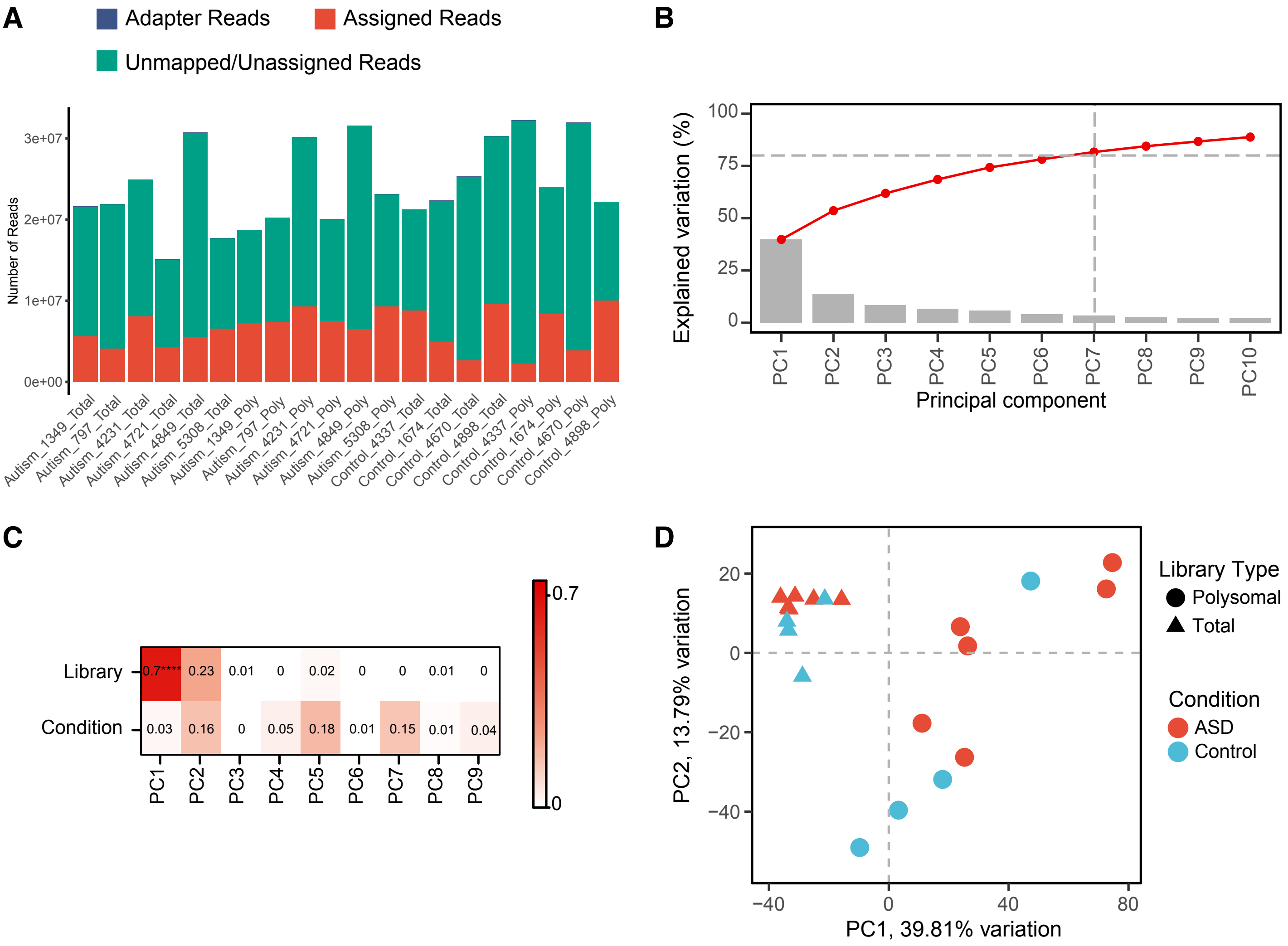

Additional Figure S3

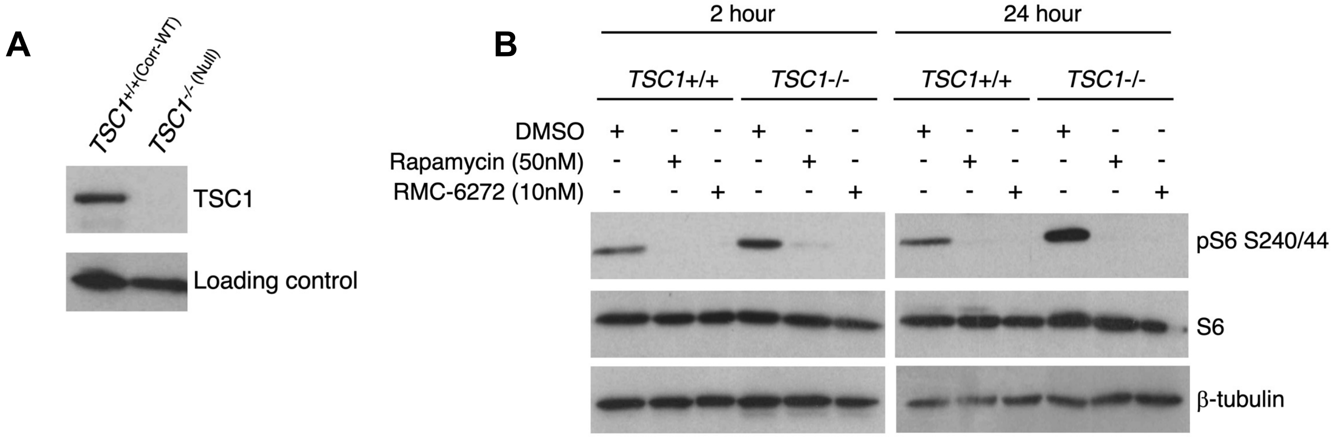

Additional Figure S4

A

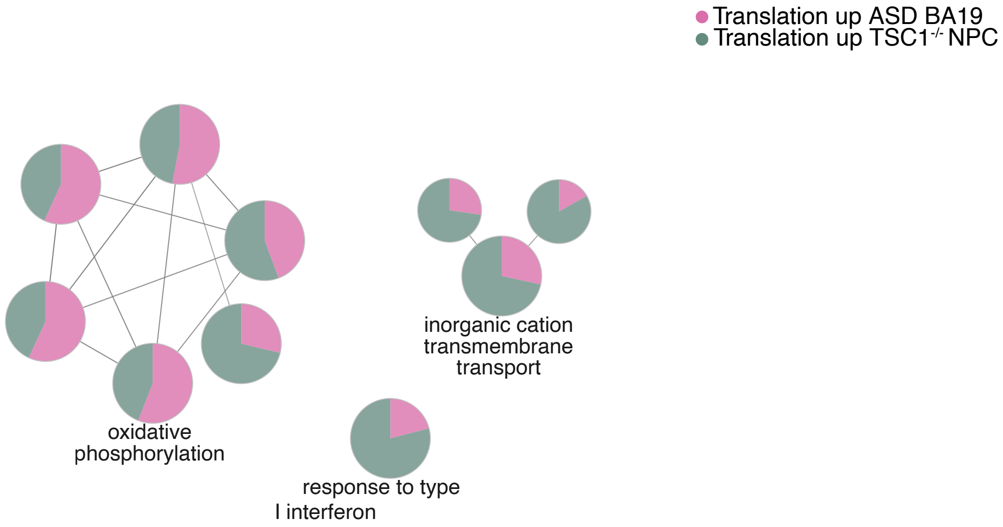

B

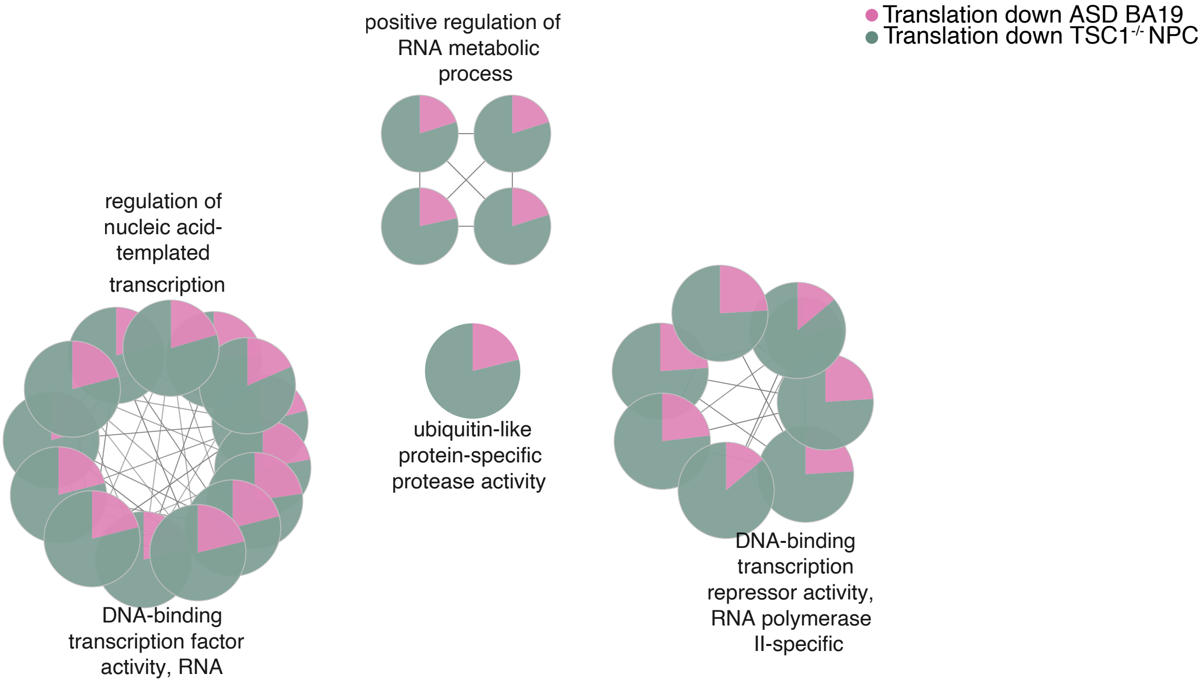

Additional Figure S5

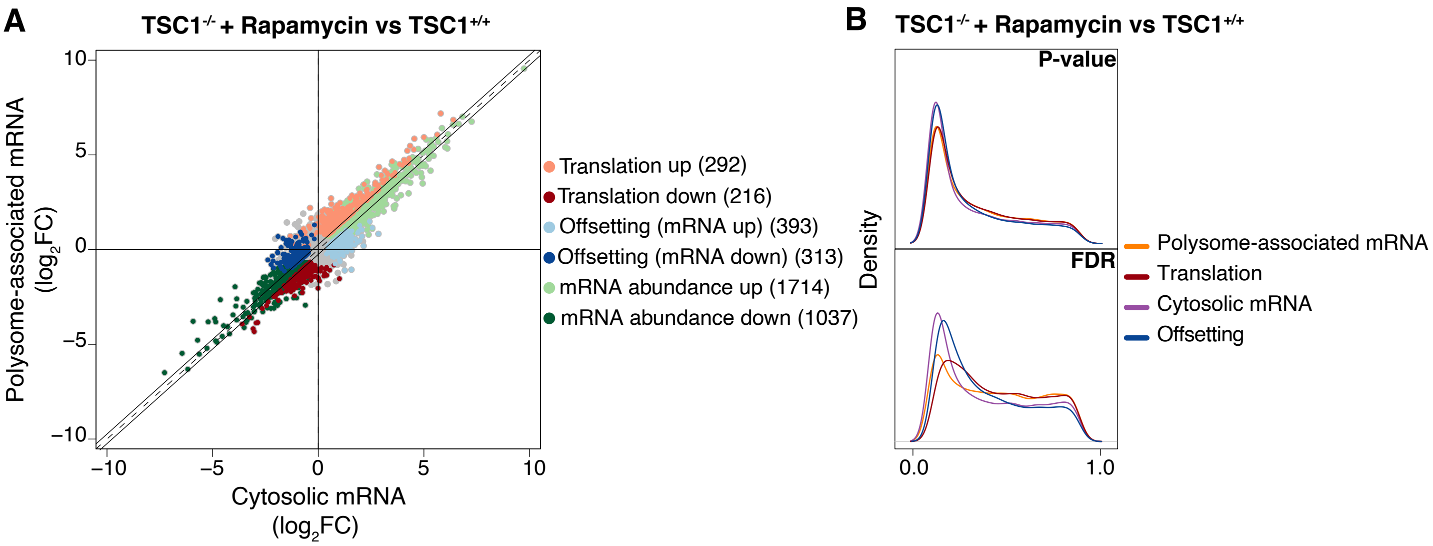

Additional Figure S6

A

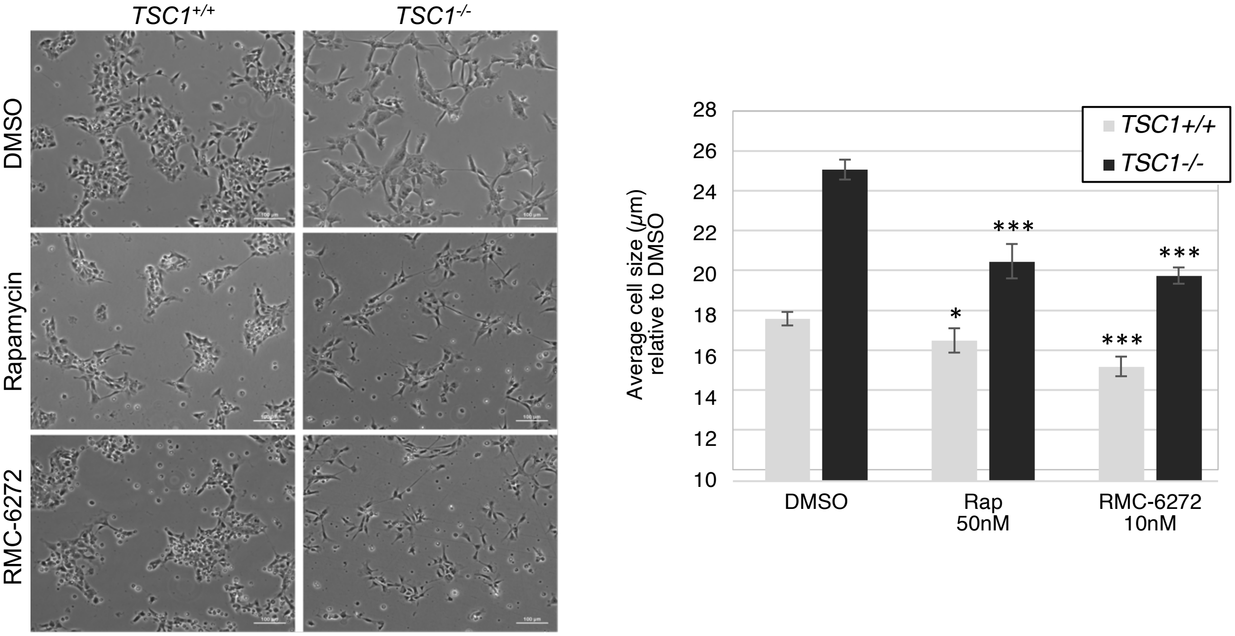

B

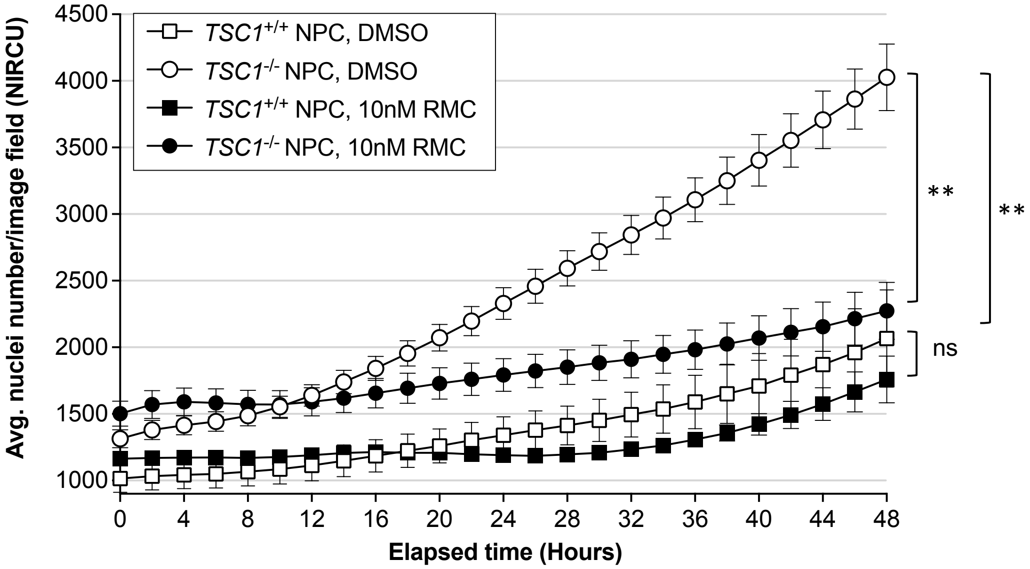

Additional Figure S7

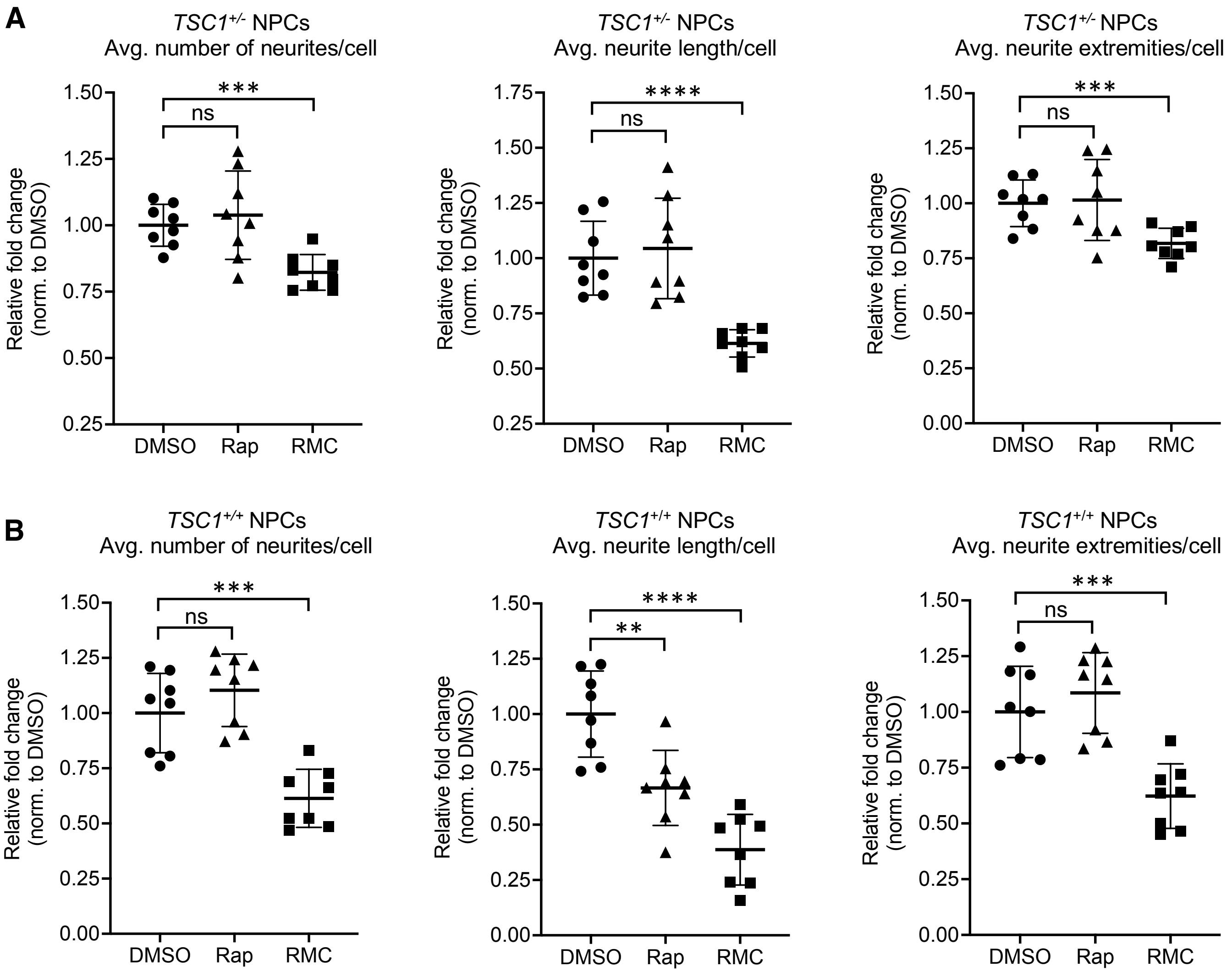

Supplement: Supplementary file 1 — Additional file 1. Figure S1. Quality control of RNA sequencing data of cytosolic and polysome-associated mRNA isolated from NPCs of different conditions. A Barplot showing overall number of reads that are aligned to adapter sequences, rRNA sequences, assigned to genes as well as unmapped/unassigned reads. B Boxplot showing TMM-log2 normalized counts of TSC1 transcript in TSC1−/− (red) and TSC1+/+ (blue) NPCs. C Scree-plot showing the percentage of variance explained by PC1-PC10. D Correlation of principal components (PC1-PC9) to experimental factors. E Projection of samples in principal components 1 and 2, with samples shaped according to library type (circle: polysome-associated mRNA; triangle: cytosolic mRNA) and colored according to genotype of samples (TSC1−/−, red; TSC1+/+, blue). Figure S2. Quality control of RNA sequencing data of postmortem samples of BA19 ASD and control. A Barplot showing overall number of reads that are aligned to adapter sequences, assigned to genes as well as unmapped/unassigned reads. B Scree-plot showing the percentage of variance explained by PC1-PC10. C Correlation of principal components (PC1-PC9) to experimental factors. D Projection of samples in principal components 1 and 2, with samples shaped according to library type (circle: polysome-associated mRNA; triangle:cytosolic mRNA) and colored according to condition (ASD: red; Control: blue). Figure S3. Immunoblotting in NPCs. A Immunoblotting for TSC1 in TSC1−/− compared with CRISPR-corrected TSC1+/+ NPCs. Ribosomal S6 protein serves as a loading control. B Immunoblot of NPCs treated with rapamycin (50 nM) and RMC-6272 (10 nM) for indicated proteins. β-tubulin served as a loading control. Images have been cropped for clarity and conciseness, and entire blots are shown in Additional file 9. Figure S4. Gene ontology analysis comparison for ASD and TSC1−/− NPCs. A and B Gene ontology analysis (similar to Fig. 1F) for genes categorized as “translation up” in TSC1−/− versus TSC1+/+ an [file 13229_2023_572_MOESM1_ESM.pdf]
