# Supplementary material for: Translatome analysis of tuberous sclerosis complex 1 patient-derived neural progenitor cells reveals rapamycin-dependent and independent alterations
Source: Mol Autism. 2023 Oct 25;14:39. doi: 10.1186/s13229-023-00572-3 (PMC10601155; doi:10.1186/s13229-023-00572-3)
Supplement: Supplementary file 9 — Additional file 9. Additional figures showing full length blots for Fig. 6A and Fig. S3. [file 13229_2023_572_MOESM9_ESM.pdf]

Additional Figures - entire blots for Fig. 6A

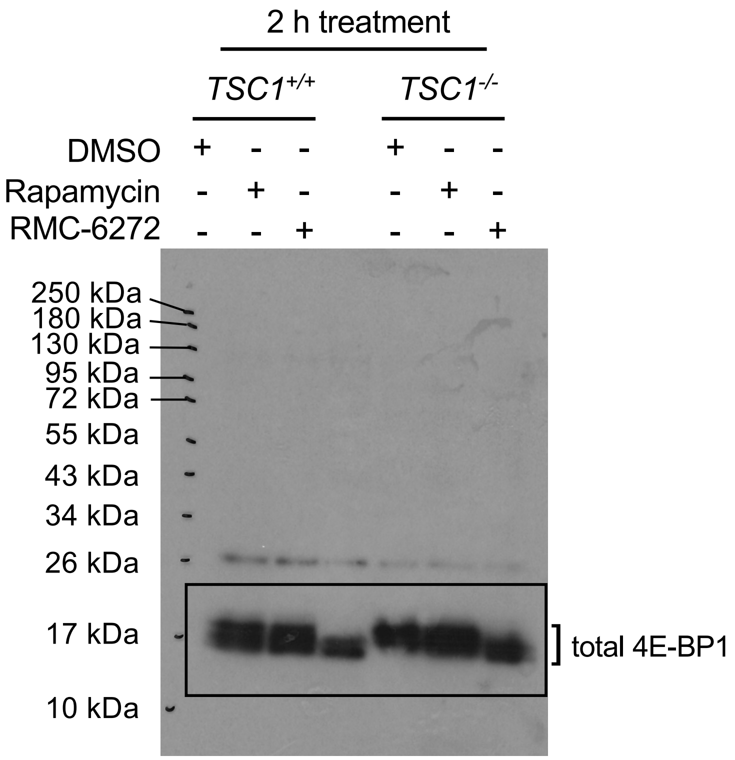

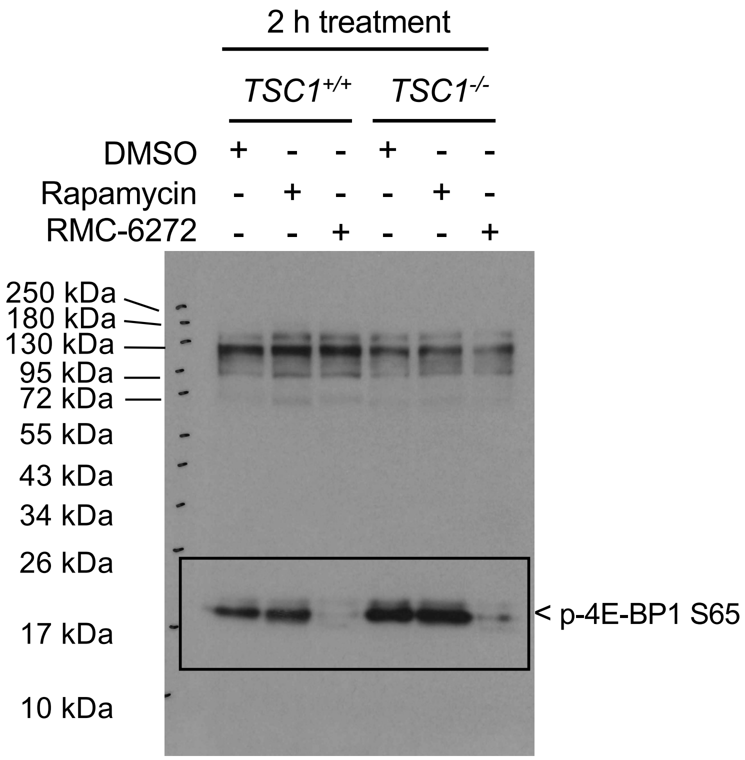

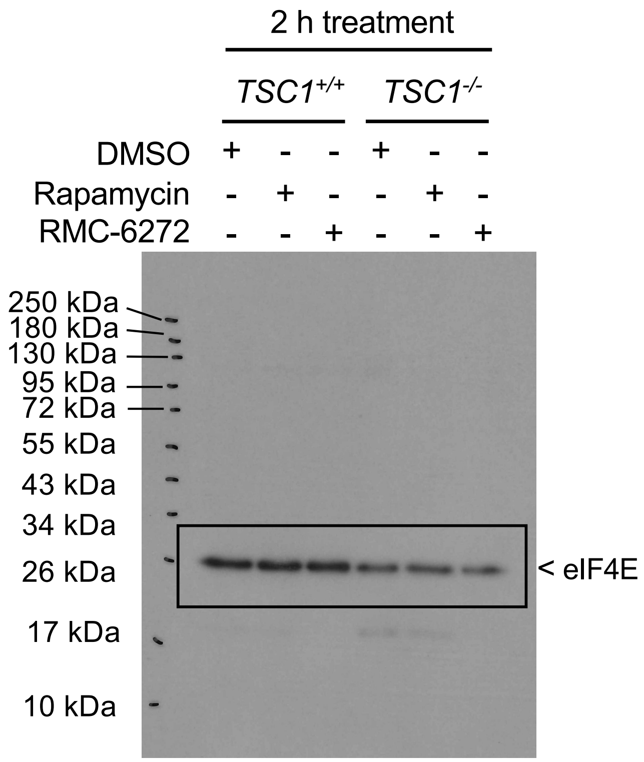

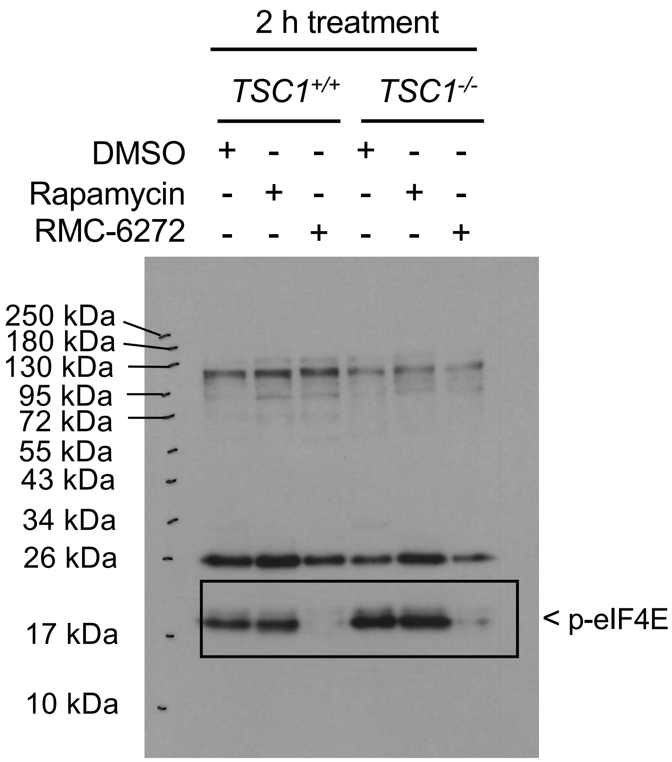

Additional Figures - entire blots for Fig. 6A

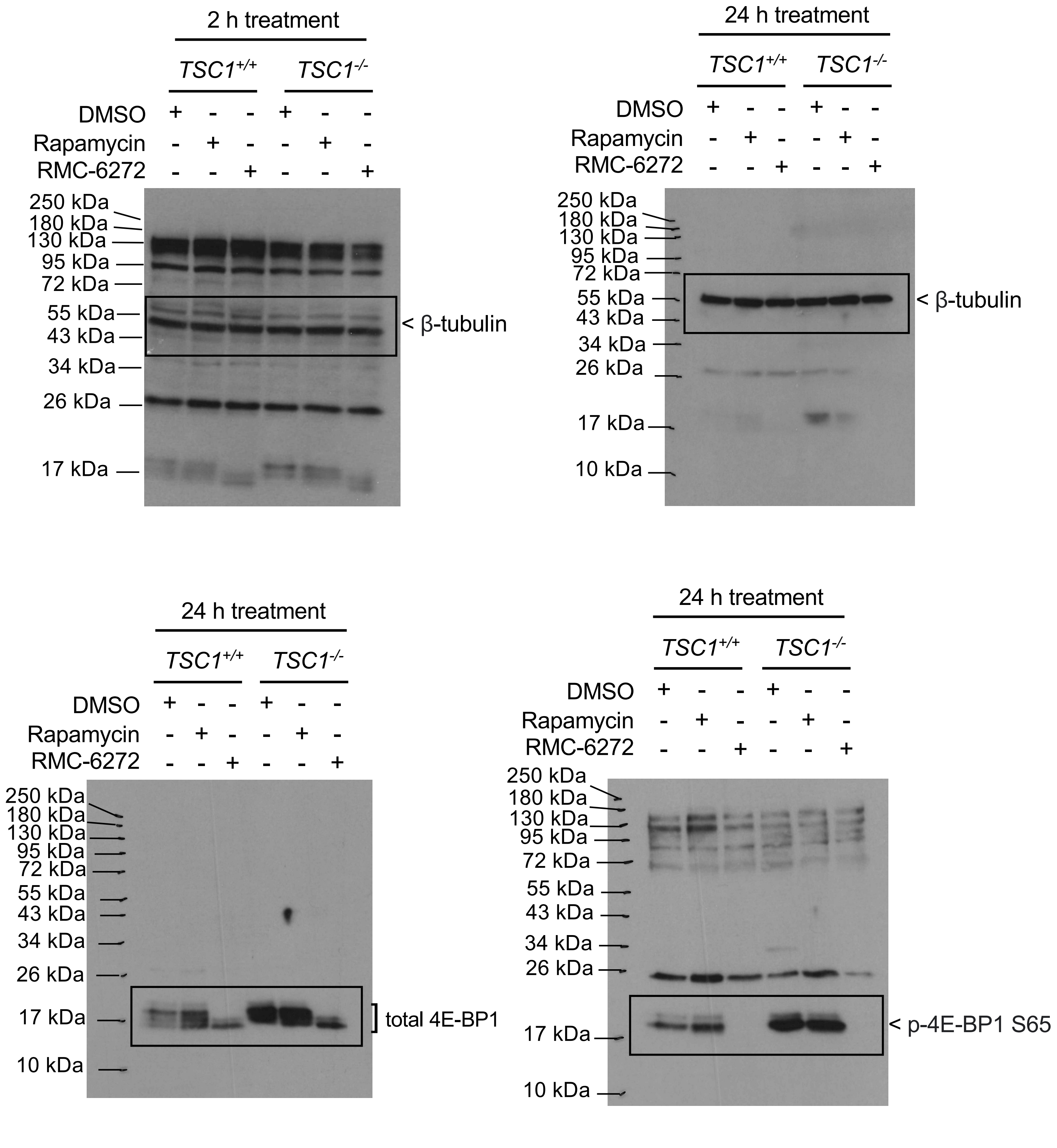

Additional Figures - entire blots for Fig. 6A

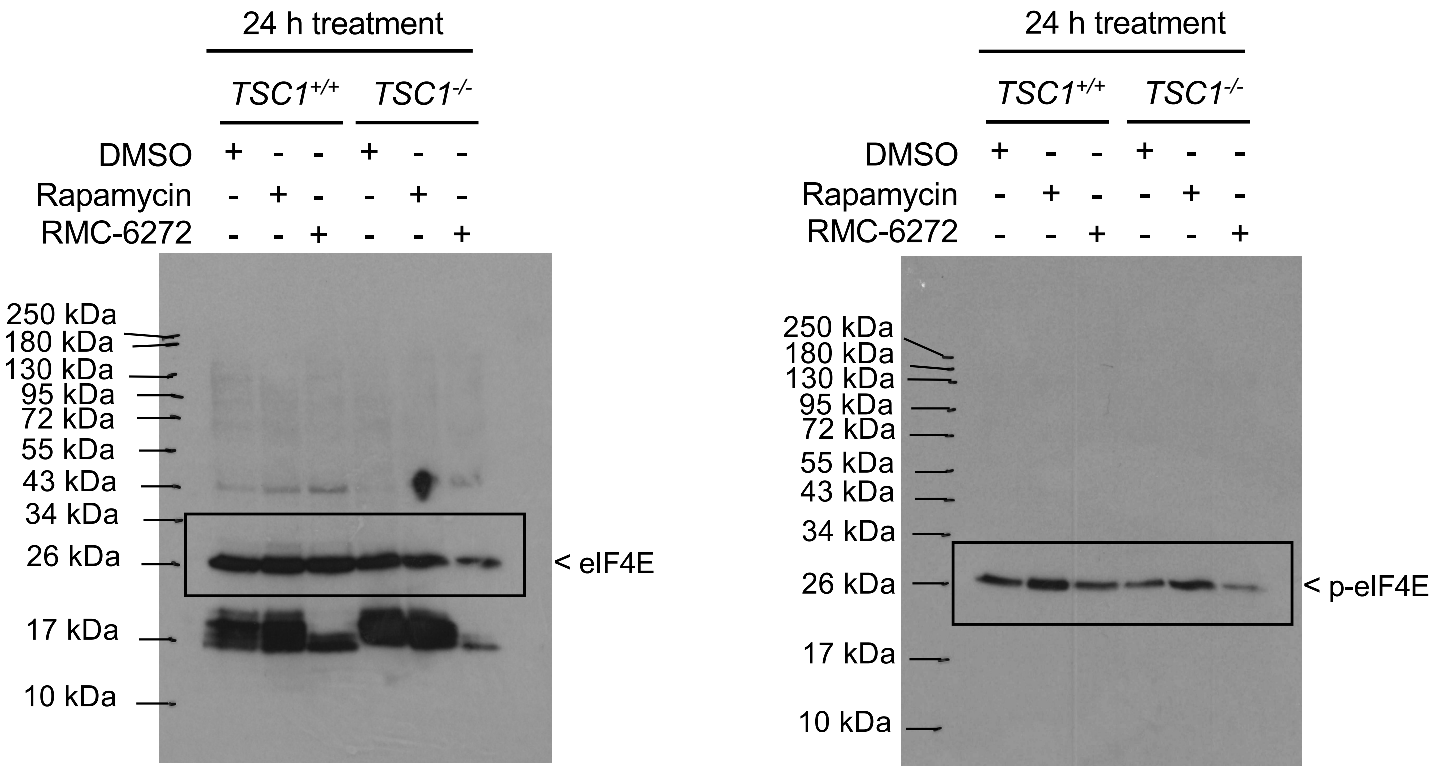

Entire blot scans related to Fig. 6A. RMC-6272 inhibits early neurodevelopmental phenotypes in TSC1-null NPCs. **A** Immunoblotting for indicated proteins in *TSC1*$^{+/+}$ and *TSC1*$^{-/-}$ NPCs treated with 10 nM RMC-6272 or 50 nM rapamycin (2 or 24h, n=3). β-tubulin serves as a loading control. Black boxes show cropped regions used in Fig. 6A.

Additional Figures - entire blots for Fig. S3A

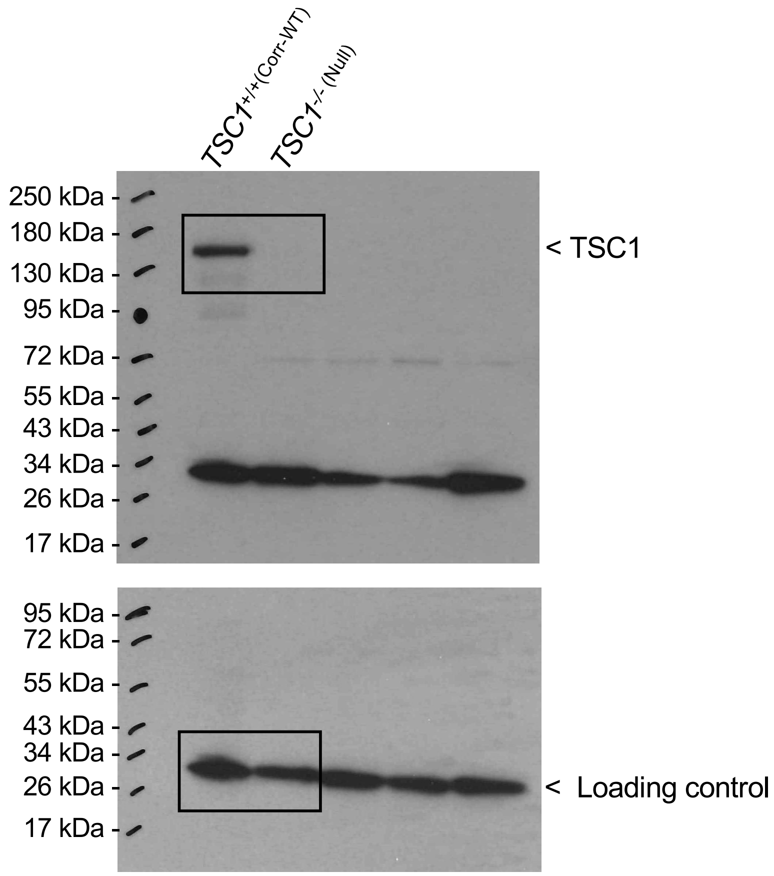

Additional Figures - entire blots for Fig. S3B

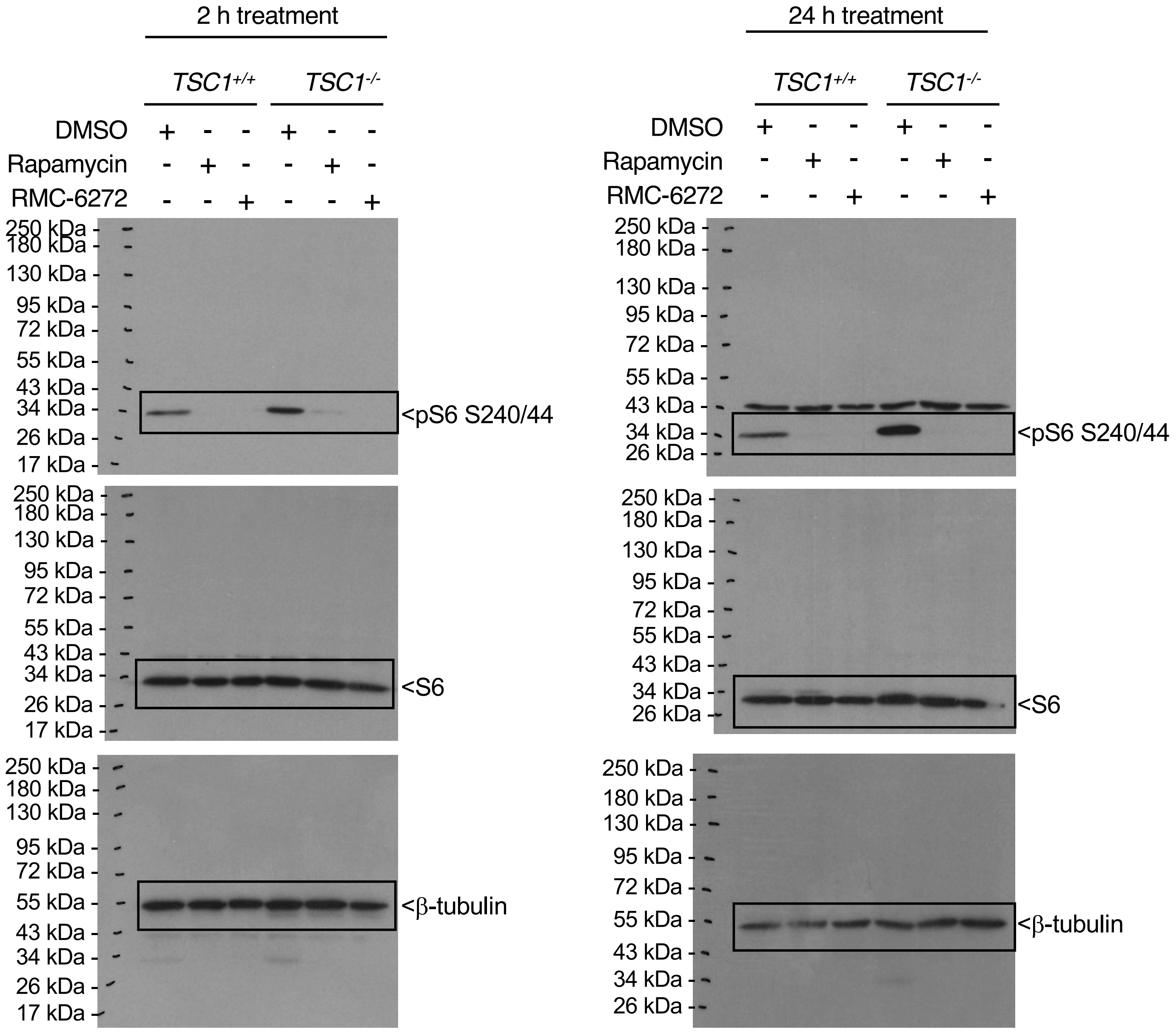

Entire blot scans related to Figure S3. Immunoblotting in NPCs. **A** Immunoblotting for TSC1 in *TSC1*$^{-/-}$ compared with CRISPR-corrected *TSC1*$^{+/+}$ NPCs. Ribsosomal S6 protein serves as a loading control. **B** Immunoblot of NPCs treated with rapamycin (50nM) and RMC-6272 (10nM) for indicated proteins. β-tubulin served as a loading control. Black boxes show cropped regions used in Fig. S3A and B
